# High Intensity Interval Training Improves Glycaemic Control and Pancreatic β Cell Function of Type 2 Diabetes Patients

**DOI:** 10.1371/journal.pone.0133286

**Published:** 2015-08-10

**Authors:** Søren Møller Madsen, Anne Cathrine Thorup, Kristian Overgaard, Per Bendix Jeppesen

**Affiliations:** 1 Department of Endocrinology and Internal Medicine, Aarhus Sygehus THG, Aarhus University Hospital, Aarhus C, Denmark; 2 Section of Sport Science, Department of Public Health, Aarhus University, Aarhus C, Denmark; University, ITALY

## Abstract

Physical activity improves the regulation of glucose homeostasis in both type 2 diabetes (T2D) patients and healthy individuals, but the effect on pancreatic β cell function is unknown. We investigated glycaemic control, pancreatic function and total fat mass before and after 8 weeks of low volume high intensity interval training (HIIT) on cycle ergometer in T2D patients and matched healthy control individuals. Study design/method: Elderly (56 yrs±2), non-active T2D patients (n = 10) and matched (52 yrs±2) healthy controls (CON) (n = 13) exercised 3 times (10×60 sec. HIIT) a week over an 8 week period on a cycle ergometer. Participants underwent a 2-hour oral glucose tolerance test (OGTT). On a separate day, resting blood pressure measurement was conducted followed by an incremental maximal oxygen uptake ($\dot{\text{V}}$O_2max_) cycle ergometer test. Finally, a whole body dual X-ray absorptiometry (DXA) was performed. After 8 weeks of training, the same measurements were performed. Results: in the T2D-group, glycaemic control as determined by average fasting venous glucose concentration (p = 0.01), end point 2-hour OGTT (p = 0.04) and glycosylated haemoglobin (p = 0.04) were significantly reduced. Pancreatic homeostasis as determined by homeostatic model assessment of insulin resistance (HOMA-IR) and HOMA β cell function (HOMA-%β) were both significantly ameliorated (p = 0.03 and p = 0.03, respectively). Whole body insulin sensitivity as determined by the disposition index (DI) was significantly increased (p = 0.03). During OGTT, the glucose continuum was significantly reduced at -15 (p = 0.03), 30 (p = 0.03) and 120 min (p = 0.03) and at -10 (p = 0.003) and 0 min (p = 0.003) with an additional improvement (p = 0.03) of its 1^st^ phase (30 min) area under curve (AUC). Significant abdominal fat mass losses were seen in both groups (T2D: p = 0.004 and CON: p = 0.02) corresponding to a percentage change of -17.84%±5.02 and -9.66%±3.07, respectively. Conclusion: these results demonstrate that HIIT improves overall glycaemic control and pancreatic β cell function in T2D patients. Additionally, both groups experienced abdominal fat mass losses. These findings demonstrate that HIIT is a health beneficial exercise strategy in T2D patients.

**Trial Registration:**

ClinicalTrials.gov NCT02333734 http://clinicaltrials.gov/ct2/show/NCT02333734

## Introduction

Type 2 diabetes (T2D) can be defined as a bihormonal metabolic disorder characterised by insufficient insulin secretion and abnormal glucagon secretion [1]. According to International Diabetes Federation, estimated worldwide prevalence of diabetes was 382 million people in 2013 with a projection of 592 million people suffering from diabetes in 2030. It is well established that physical activity *per se* improves glucose homeostasis [2–4], a cornerstone of regulating overall glycaemic control among T2D patients. As of 2013, it is recommended that T2D patients should perform at least 150 minutes per week of moderate-intensity aerobic exercise corresponding to 50–70% of maximal heart frequency [5,6]. Since Bjorntorp and co-workers established the importance of regular moderate to strenuous exercise to increase insulin sensitivity among T2D patients [7], considerable molecular and metabolic research has demonstrated pivotal (patho)physiological linkages between health-related benefits of physical activity and T2D. Though physical exercise is advocated in the treatment of T2D, existing strategies face huge challenges, including the lack of adherence, motivation and time to follow these guidelines [6].

Recently, more focus has addressed the health beneficial effects of different high intensity interval training (HIIT) regimens to T2D. Novel findings here include reduced hyperglycaemia following 2 weeks of HIIT on cycle ergometer [8,9], ameliorated insulin action and upregulated skeletal muscle metabolic capacity after walking intervals [10] and improved pancreatic β-cell function [11]–pivotal physiological phenomena in the attempt to regulate whole body metabolism in T2D patients. These health beneficial effects seem to be independent of changes in body weight in moderate intensity training regimens [12–14], and glycaemic control may be even more improved by intensities above recommended guidelines [15,16]. There seems to be accumulating evidence that HIIT induces increased overall fat loss and abdominal fat mass loss as opposed to traditional continuous endurance training [17]. The cardiovascular adaptations that appear with HIIT are comparable, or even sometimes superior as opposed to traditional continuous endurance training [18–20]. However, in the T2D population, research in long-term training-induced changes of both glycaemic control and the pancreatic homeostasis is sparse and further detailed knowledge on this topic is needed to evaluate the true clinical effect of training.

In inactive individuals diagnosed with T2D and age- and BMI-matched normal glucose tolerant subjects, we therefore tested the effects of 8 weeks of low volume HIIT on 1) Glycaemic control (fasting plasma glucose concentration, 2-hour oral glucose tolerance test (OGTT) response, area under curve (AUC) and glycosylated haemoglobin (HbA_1C_)), 2) pancreatic homeostasis (OGTT-derived surrogate markers) and 3) total fat and abdominal fat mass.

## Methodology

### Subjects

**Fig 1:**
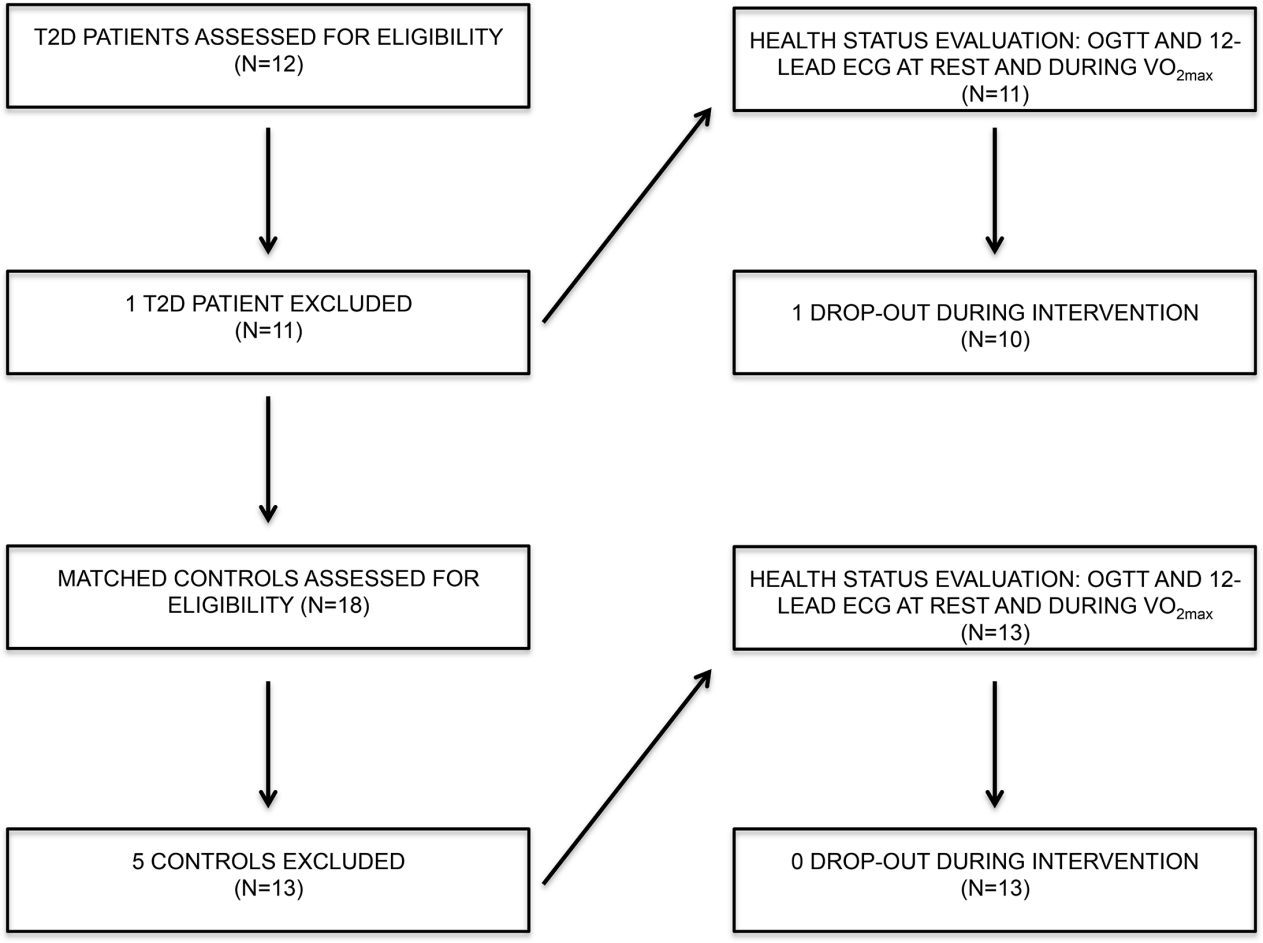
Flow chart. 1 T2D patient was excluded due to impossible insertion of catheter, and the last T2D patient dropped out of the study immediately after the initiation of HIIT. 5 matched controls were excluded due low BMI.

Subjects were recruited through local newspaper advertisements and evening sessions at Centre for Clinical Research, Vendsyssel Hospital, Aalborg University, where the study was carried out. 10 T2D patients and 13 age, height and weight matched control (CON) individuals were eligible for the study (see flow chart, Fig 1). All individuals answered a medical questionnaire to evaluate their individual lifestyles (history of genetic T2D, physical activity, smoking habits, alcohol consumption and diet). T2D inclusion criteria were: 2-hour end point OGTT ≥ 11.1 mmol·l^-1^, BMI < 40 kg·m^-2^, both genders and < 65 of age. Exclusion criteria were: diabetes duration < 1 year, BMI < 25 kg·m^-2^, moderate intensity exercise > 1 hour per week, use of exogenous insulin, evidence of liver, renal, cardiopulmonary, neuromuscular and/or psychological disease, other debilitating diseases or contraindicating physical activity [21]. Furthermore, there were two eligibility tests during the visits: 1) if markers or analytes as given below in detail did not fulfil criteria ranges, subjects were excluded and 2) if there were any perturbations during the heart cycle electrocardiogram (ECG) of both resting and working myocardium, subjects were excluded. All ten T2D patients were under treatment with oral antidiabetic agents, either with metformin (N = 8) or glimepiride (N = 2). Additionally, lipid-reducing agents (N = 9), anti-hypertensive agents (N = 8), glucagon-like peptide-1 (GLP-1) receptor agent (N = 1) and glucagon-like peptide-1 (GLP-1) inhibitor agent (N = 3) were taken on a daily basis. All subjects continued their medication throughout the study.

The study was approved by the Ethics committee of Central and North Denmark Region (M-2013-56-13) and implemented in accordance with the guidelines of *Declaration of Helsinki*. The study was registered at www.ClinicalTrials.gov. (NCT02333734). Written informed consent was obtained from all participants prior enrolment to the study.

Participants were carefully instructed to continue their individual eating habits and to maintain a eucaloric diet [22].

### Experimental overview

**Fig 2:**
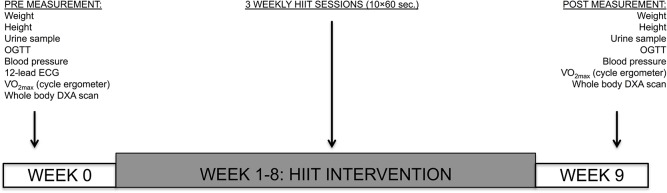
Study overview of the experiment prior to and after the HIIT intervention. On a separate day, a urine sample, oral glucose tolerance test (OGTT), weight and height measurements were conducted. On another day: resting blood pressure measurement in supine position and 12-lead electrocardiogram (ECG) prior and after measurement of maximal oxygen consumption on bicycle ergometer. On the last experimental day, a whole body dual energy X-ray absorptiometry (DXA) was performed. These measurements were followed by 8 weeks of HIIT. Finally, laboratory measurements were repeated.

Baseline post intervention outcomes were assessed over three test days in the week before and in the week following the HIIT program. On Mondays, Wednesdays and Fridays in week 1–8, participants conducted HIIT. An outline of the experimental time schedule appears in Fig 2.

### Study design

#### Day 1 –oral glucose tolerance test

All participants underwent an OGTT before (week 0) and again after the HIIT intervention (week 9). All subjects abstained from performing any strenuous physical activity for 2–3 days prior to both OGTTs to avoid the effects of acute exercise. The participants came into the clinic after a 12-hour overnight fast. Before the initiation of the OGTT, a fasting urine sample was collected from each participant and was immediately frozen and stored at -80°C. This was followed by an assessment of body weight (TANITA, BWB-800), height (only at week 0) and waist circumference in duplicate at the level of the umbilicus conducted by a trained person. Then fasting capillary blood glucose was conducted and analysed in duplicate using an OneTouch glucose monitoring apparatus (Precision Xceed MediSense, Abbott Laboratories A/S, Denmark).

Afterwards, a cannula (Beckton, Dickinson & Company, Franklin Lakes, NJ, USA) was carefully placed into an antecubital vein followed by infusion of saline solution to control and maintain clean access. Fasting venous blood samples were taken to the times -15, -10 and 0 minutes before a glucose load of 75 g anhydrous glucose dissolved in water was given. At the first (-15 min) fasting venous blood sample, 4 blood samples were taken to screen the coagulation (II, VII, X and INR), liver (ASAT and ALAT), cell (leucocytes, erythrocytes and thrombocytes), kidney (albumin and creatinine), inflammatory (CRP) and hormonal (TSH) status to preclude any underlying disease. At same time point (-15 min), additional 3 blood samples were taken to determine overall inflammation, lipids and HbA_1C_. At all time points (-15, -10 and 0 min), a blood sample was collected to analyse glucose, insulin and glucagon. Following the glucose load ingestion, venous blood samples were taken after 15, 30, 60 and 120 minutes and were assayed for glucose, insulin and glucagon concentrations. ~10 g of saline solution was flushed through the vein after each collected blood sample. Each blood sample was transferred to either 2.7 ml EDTA (Beckton, Dickinson & Company, Franklin Lakes, NJ, USA) or lithium heparin (Beckton, Dickinson & Company, Franklin Lakes, NJ, USA) evacuated tubes, briefly stored on ice (<30 min) and centrifuged at 4°C for 15 min at 3000 rpm to provide plasma samples. Plasma was then separated into aliquots and stored at -80°C until further analysis.

#### Biochemical analyses

All analytic processes were conducted according to the manufacturer’s instructions. Plasma glucose content (unit: mmol·l^-1^) was determined applying an enzymatic reference method (Roche Diagnostics, Switzerland) on a Cobas c111 system. Plasma insulin concentrations (unit: pmol·l^-1^) were measured with an enzyme-linked immunosorbent assay (ELISA) (Dako, Glostrup, Denmark) and plasma glucagon concentrations (unit: pg·ml^-1^) were determined by a radioimmunoassay (RIA) kit (EMD Millipore Corporation, Billerica, MA, USA). Glycosylated haemoglobin (HbA_1C_) was determined applying high-performance liquid chromatography (HPLC) on the TOSOH G8 (Tosoh Bioscience, Tokyo, Japan). Health status markers, fasting total cholesterol (TC), high-density lipoprotein (HDL) and triglyceride (TG) were determined using the immunonephelometry procedure (Siemens, Erlangen, Germany) on the Vista Dimension 1500 system. Low-density lipoprotein (LDL) was estimated by using the Friedewald equation. Except for glucose, all samples were analysed in duplicate.

#### Day 2 –aerobic testing and blood pressure

On a separate day, subjects performed an incremental exercise test on a cycling ergometer (Monark 928 E, Varberg, Sweden) to determine maximal pulmonary oxygen uptake ($\dot{\text{V}}$O_2max_). The exercise protocol began with a warm-up phase of 8 minutes at a workload of 80–100 W, inducing a working heart frequency of ~65% of the estimated maximal heart rate (Polar 625X, Kempele, Finland). A facemask was fitted covering mouth and nose. The mask was instrumented with a flowmeter and a sampling tube allowing for continuous breath-by-breath measurement of ventilation volume and expired O_2_ and CO_2_ concentrations. These data were used for calculation of $\dot{\text{V}}$O_2_ values (Oxycon Pro, Jaeger Gmbh, Hoechberg, Germany). After careful instruction, each subject performed the $\dot{\text{V}}$O_2max_ test with a workload that was increased by 15 W·min^-1^. During the last minutes of the test, the subjects were vigorously encouraged to continue until exhaustion, and the achievement of $\dot{\text{V}}$O_2max_ was established by standard criteria in all tests [23]. The gas analyzers and the flow were calibrated prior to each test. Individual power output used for the HIIT intervention was taken as the power output eliciting ~90% of maximal heart rate during the $\dot{\text{V}}$O_2max_ test. The test was repeated in week 9 after the completion of the HIIT intervention.

Subjects had their resting and post-work heart function controlled by 12-lead electrocardiogram (ECG) (Mortara ELI 150, Milwaukee, WI, USA). There were no signs of ischemia or arrhythmias during rest or after the $\dot{\text{V}}$O_2max_ test as controlled by a physician.

Prior to each $\dot{\text{V}}$O_2max_ test, blood pressure (Microlife BP A100 Plus, Widnau, Switzerland) was registered in a supine position in a quiet room at ambient temperature. The cuff was placed around the participant’s left arm and inflated every 10 minutes (0–30 minutes). Systolic (BPs) and diastolic (BPs) were registered followed by measurement of mean arterial pressure (BPm), i.e. BPd+⅓×(BPs-BPd). The lowest values were used for comparison. There was no or little (<30 min) intra-individual variability in the time points when the parameters were assessed before and after the HIIT intervention.

#### Day 3

On a separate day (week 0), a whole body dual x-ray absorptiometry (DXA) scan (XR-800 Swissray, Cooper, Surgical, USA) was conducted at Frederikshavn Hospital. Additionally, abdominal and femoral regions were assessed for fat mass and lean mass. Another DXA scan (week 9) was conducted after the completion of the HIIT intervention for comparison. A trained radiographer performed all scans.

### High intensity interval training program

Subjects conducted supervised HIIT on a cycling ergometer (Kettler Axiom, Germany) three times per week over 8 weeks (every Monday, Wednesday and Friday) in three groups of five and two groups of four. During the entire intervention, a physiotherapist supervised the subjects to control that they adhered to and completed their individual power output obtained from their $\dot{\text{V}}$O_2max_ test. Halfway through the intervention subjects had their power output increased by ~5% for the remaining period in order to maintain a sufficient relative intensity despite neural and muscular adaptations. Each training session consisted of a 5-minute warm-up (eliciting ~65% of HR_max_) followed by 10×1 minute intervals each interspersed by 1 minute of recovery [9,24]. Cadence during the HIIT was ~70 RPM. During recovery, the participants were allowed to either rest or pedal with a minimum of resistance. The session was completed by a cool-down period of 5 minutes. Total time commitment was ~30 minutes per HIIT session. During each training session, the heart rate was registered from the cycling ergometer with additional reporting of rating of perceived exertion (Borg Scale). During the intervention, participants were only allowed to exercise during the HIIT sessions. However, we allowed easy gardening, walking and other daily activities.

### Calculations and statistical analyses

Statistical analyses were conducted applying the STATA 13 statistical analysis system (StataCorp LP, Texas, USA), whereas the GraphPad Prism 6 statistical analysis system (GraphPad Software Inc., San Diego, CA, USA) was applied for visual presentation. In the visual presentation, mean ± S.E.M. are given. All data were tested for normality applying QQ-plots and Bland Altman Plots. After careful evaluation, all data fulfilled the criteria of being normally distributed. *Student’s paired t-test* was applied to test possible differences within same group before and after the HIIT intervention. Also, *student’s paired t-test* was applied to determine possible changes between same time points during the OGTT before (week 0) and after the HIIT intervention (week 9). AUC was calculated by the integrated trapezoid rule [25]. The insulin resistance homeostatic model assessment (HOMA-IR) was determined from fasting plasma glucose (FPG) and fasting plasma insulin (FPI) concentrations by ((FPG×FPI)/22.5). The β-cell function (HOMA-%β) was calculated by ((20×FPI)/(FPG-3.5)). Applied glucose unit: mmol·l^-1^, applied insulin unit: μIU·ml^-1^ (determined as pmol·l^-1^/6) [26]. Composite insulin sensitivity index (ISI_composite_) was calculated according to the formula [27]: 10.000 divided by the square root of ((FPG×FPI)×(mean FPG×mean FPI). Insulin secretion was assessed by the insulinogenic index (IGI) [28] determined from the ratio of increases of plasma insulin to glucose measured at 30 minutes expressed as: ΔI30/ΔG30, i.e. (Insulin 30 min—Insulin 0 min)/(Glucose 30 min–Glucose 0 min). The product of the ISI_composite_ and the IGI, the so-called disposition index (DI), is a useful marker of pancreatic β-cell function [29]. FPG and FPI were calculated on the basis of all fasting blood samples, i.e. the mean of -15, -10 and 0 min. Applied units were the same as already given above. In all cases, p<0.05 was considered significant. In each patient category, at least 10 individuals were mandatory to be included to achieve statistical power of metabolic parameters with 80% power and with a significance level, α = 0.05.

## Results

During the 8-week HIIT intervention both T2D and CON groups showed similar high training compliance (heart rate at end HIIT bout was ~90% of maximal heart rate) and there was total adherence from both groups in all 8 weeks. Except for 1 dropout, all subjects participated in the full range of the supervised 8-week HIIT sessions without exception, which highlights the feasibility of this exercise regimen.

### Glycaemic control

**Fig 3:**
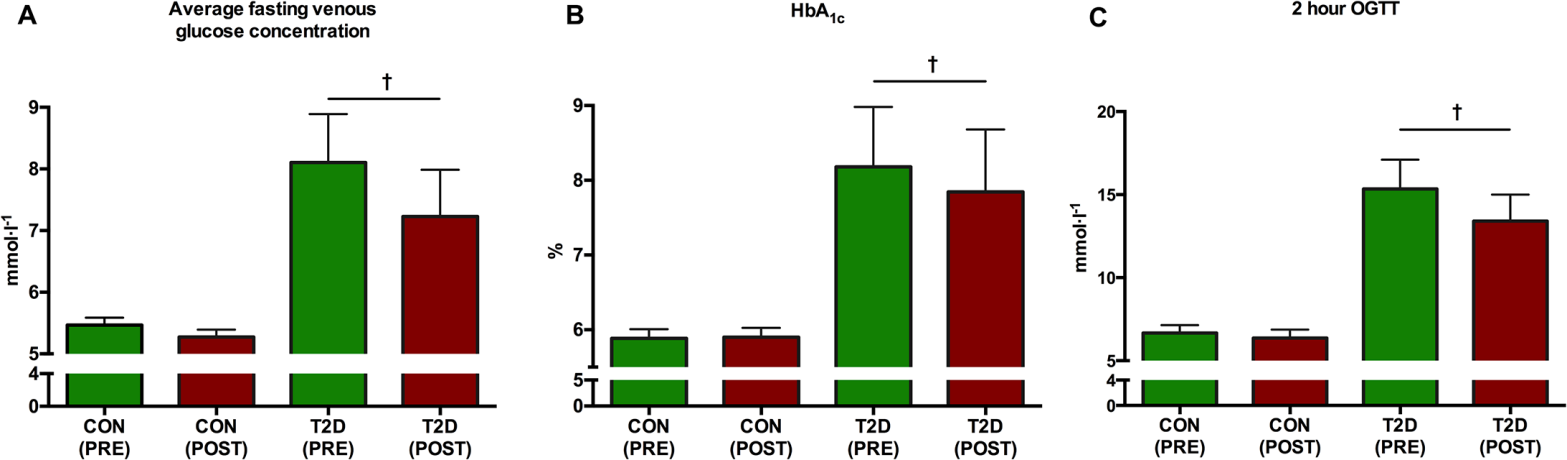
The development of glycaemic control prior to and after the HIIT intervention. In the CON-group, there were no statistical effects (p>0.05) observed on average fasting venous glucose concentration (A), HbA_1C_ (B) or postprandial glucose concentration 2 hours after OGTT (C). However, following 8 weeks of low volume HIIT, statistical significant reductions were detected on average fasting venous glucose concentration (A), HbA_1C_ (B) and postprandial glucose concentration 2 hours after OGTT (C) in the T2D-group (all denoted by †).

In the CON-group, no significance was observed in either average fasting venous glucose concentration (p = 0.17), the 2-hour OGTT levels (p = 0.58) or HbA_1C_ (p = 0.81). Among the T2D patients, significant reductions of average fasting venous glucose concentration (p = 0.01) (p = 0.0106), HbA_1C_ (p = 0.036) and 2-hour end OGTT levels (p = 0.035) were observed (Fig 3A, 3B and 3C).

### OGTT-derived indices to assess pancreatic β-cell function and insulin resistance

**Fig 4:**
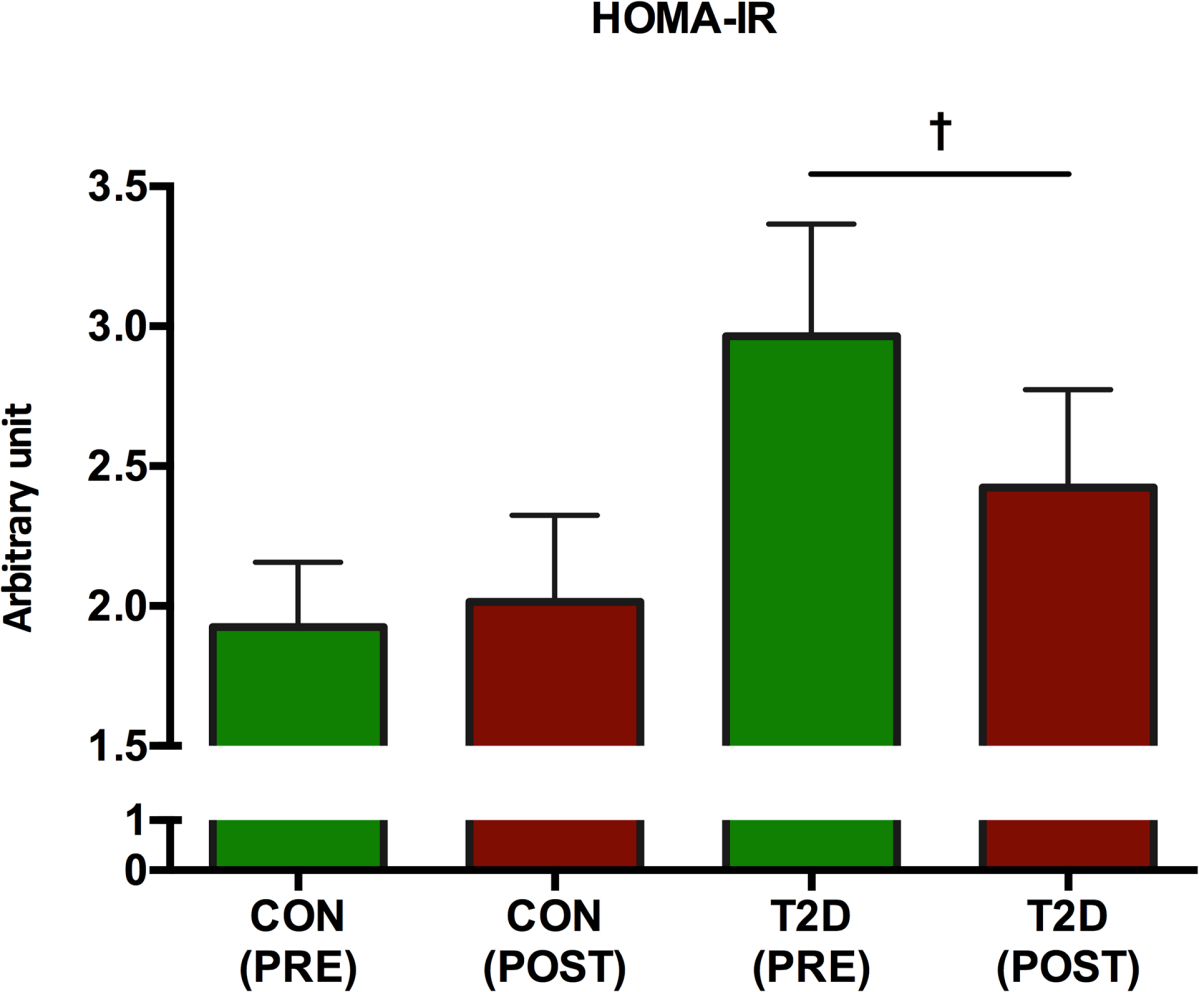
The development of HOMA-IR. Following 8 weeks of HIIT, the HOMA-IR in the CON-group remained unchanged (p>0.05), whereas HOMA-IR was significantly reduced p = 0.035 in the T2D-group (as denoted by †).

**Fig 5:**
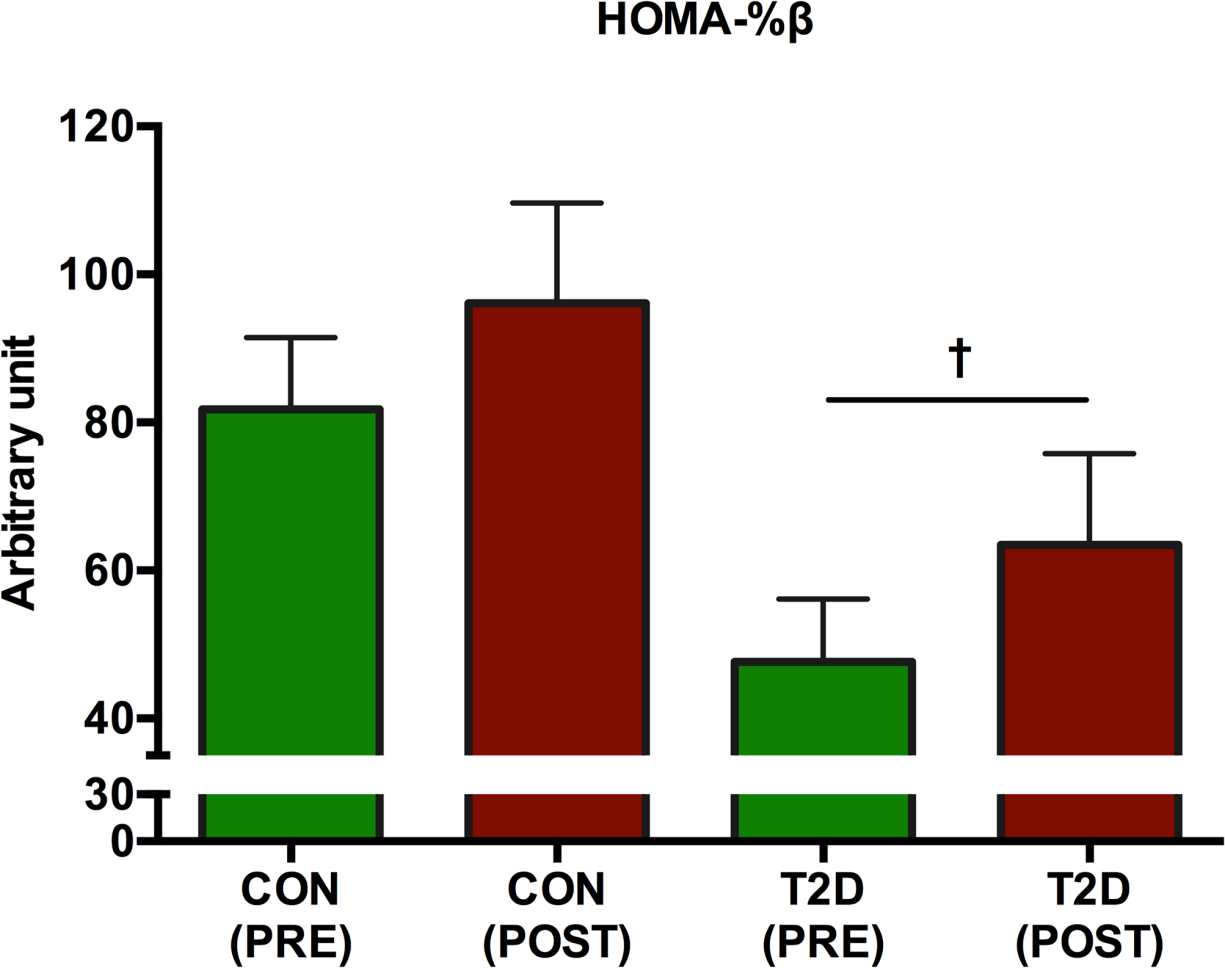
The development of HOMA-%β. Following 8 weeks of HIIT, the HOMA-%β in the CON-group remained unchanged (p>0.05), whereas HOMA-%β was significantly reduced p = 0.026 in the T2D-group (as denoted by †).

**Fig 6:**
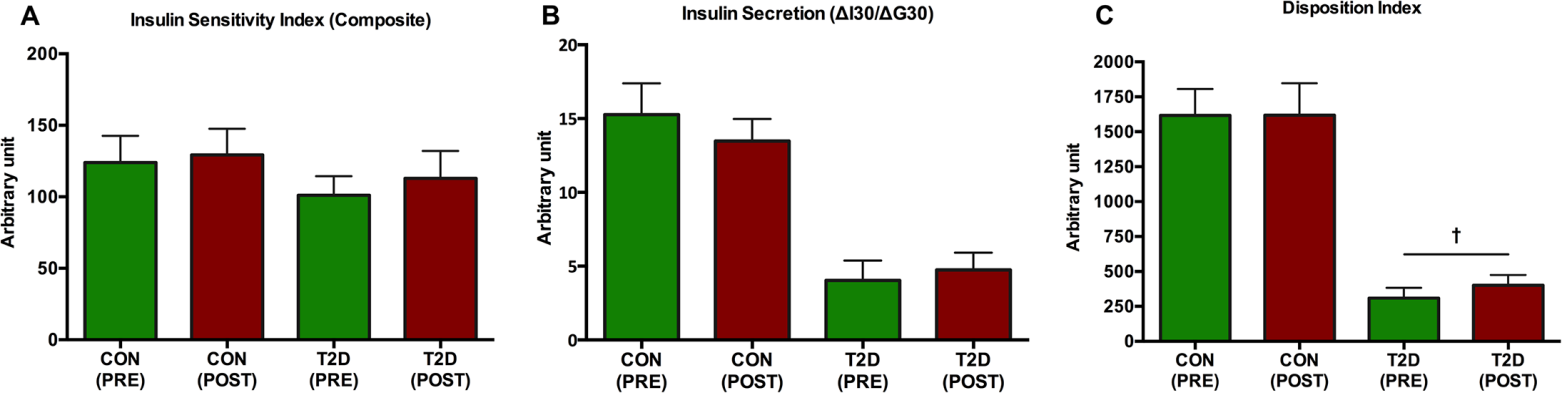
The development of ISI_composite_ (A), Insulin secretion (B) and DI (C). Following 8 weeks of HIIT, ISI_composite_, Insulin secretion and DI remained unchanged (p>0.05) in the CON-group. In the T2D-group, pancreatic β-cell function as determined by DI was significantly elevated (p = 0.03) (denoted by †). In both groups, neither ISI_composite_ nor insulin secretion was significantly reduced (p>0.05).

In the CON-group, neither HOMA-IR (p = 0.63) nor HOMA-%β (p = 0.22) was significantly changed (Figs 4 and 5, respectively). However, in the T2D-group, HOMA-IR was significantly (p = 0.03) decreased (Fig 4) and HOMA-%β was significantly (p = 0.03) increased (Fig 5). Insulin secretion as determined by the insulinogenic index and ISI_composite_ remained unaltered (p>0.05) in both groups (Fig 6A and 6B). Pancreatic β-cell function as determined by DI was unaltered in the CON-group (p = 0.99), but significantly improved (p = 0.03) in the T2D-group (Fig 6C).

**Fig 7:**
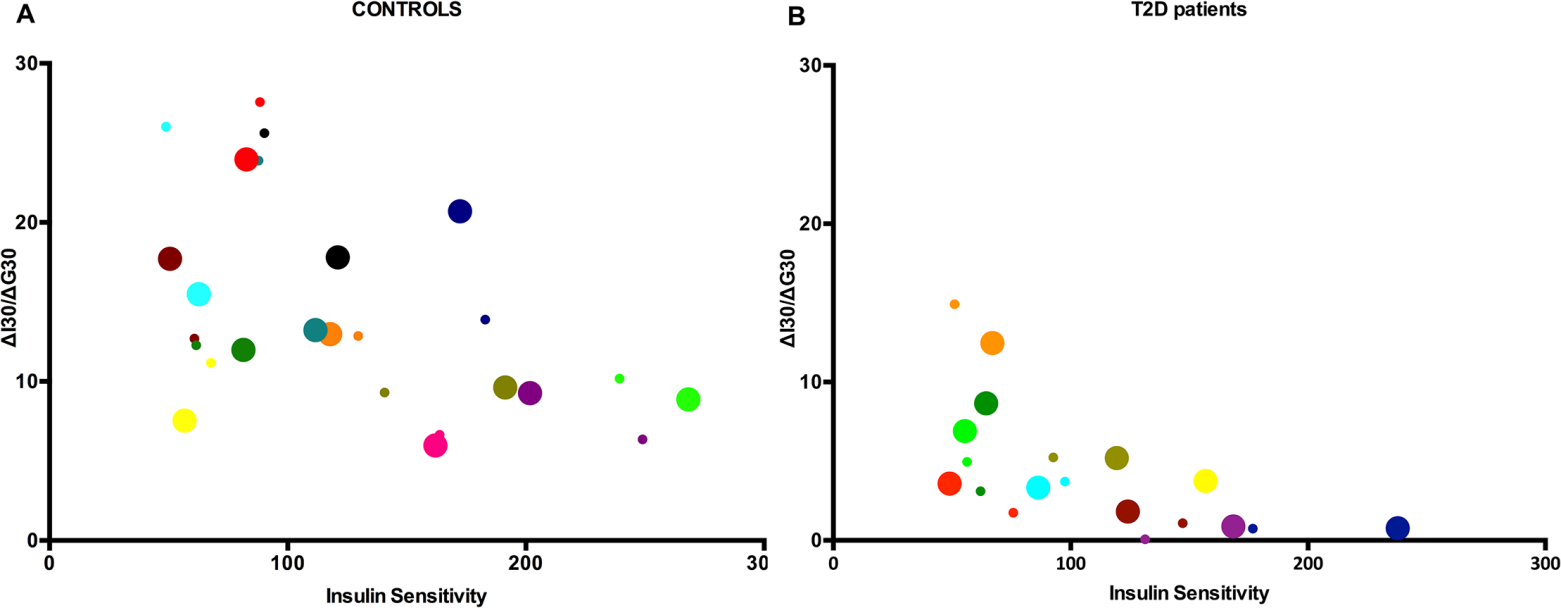
Insulin sensitivity (abscissa axis) and insulin secretion (y-axis) among controls (A) and among T2D diabetes patients (B). Small dots represent values prior HIIT intervention, whereas big dots represent values after HIIT intervention. Each colour represents one individual.

**Fig 8:**
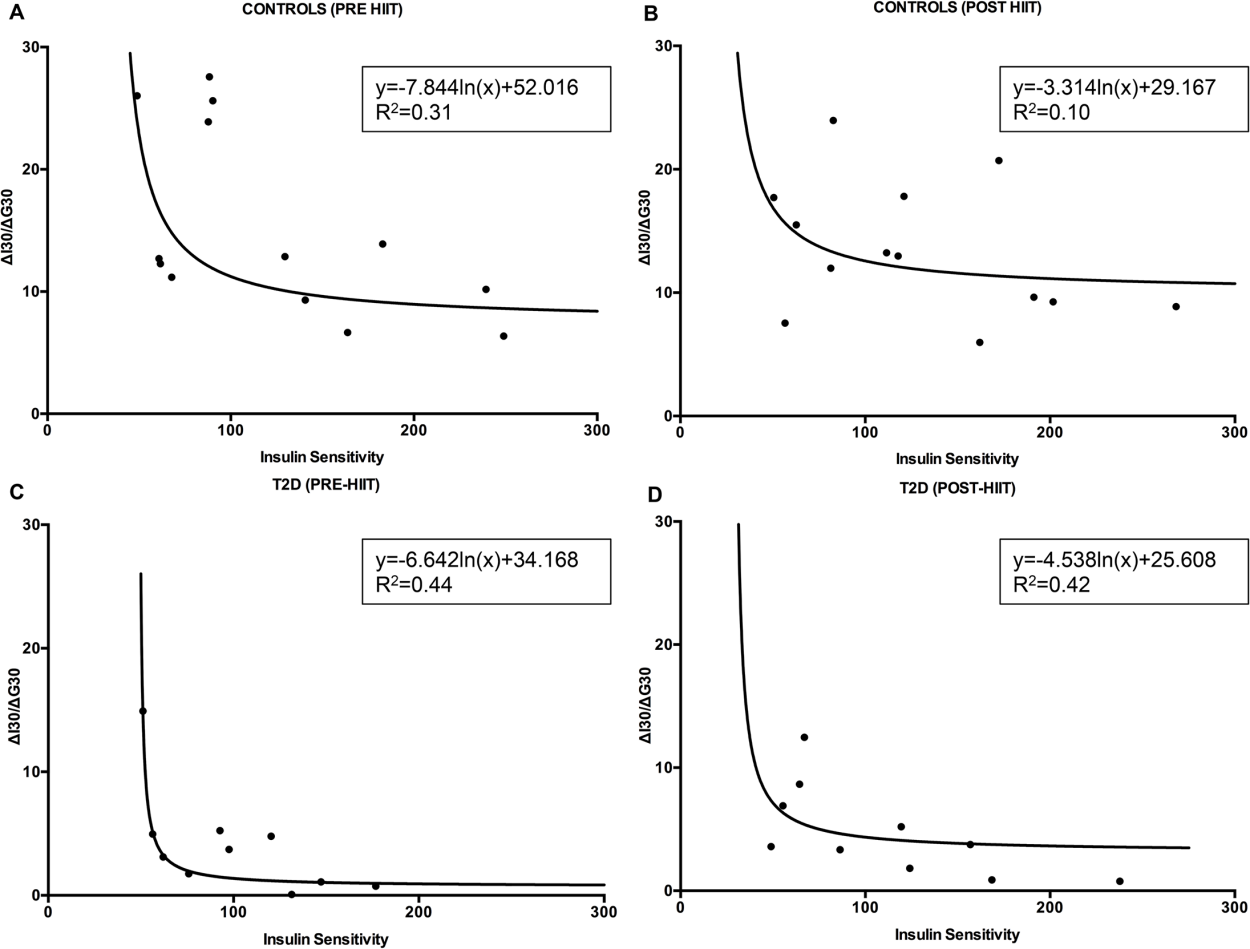
Hyperbola fitting of insulinogenic index and composite insulin sensitivity index. These differences between before and after HIIT can be seen from both the CON-group (A and B) and the T2D-group (C and D).

The hyperbolic intra-relationships between ISI_composite_ and IGI are shown in Fig 7A (CON-group) and 7B (T2D-group). Additionally, the over-all best-fit lines attained by nonlinear regression analysis (logarithmic regression) between ISI_composite_ and IGI are presented in Fig 8A–8D. The overall regression line between ISI_composite_ and IGI among T2D patients was y = -6.642ln(x)+34.168 (R^2^ = 0.44) prior the HIIT intervention (Fig 8A) and y = -4.538ln(x)+25.608 (R^2^ = 0.42) afterwards (Fig 8B). For the CON-group, the overall regression line was y = -7.844ln(x)+52.016 (R^2^ = 0.31) before HIIT (Fig 8C) and y = -3.314ln(x)+29.167 (R^2^ = 0.10) after HIIT (Fig 8D).

### Plasma glucose, insulin and glucagon concentrations

**Fig 9:**
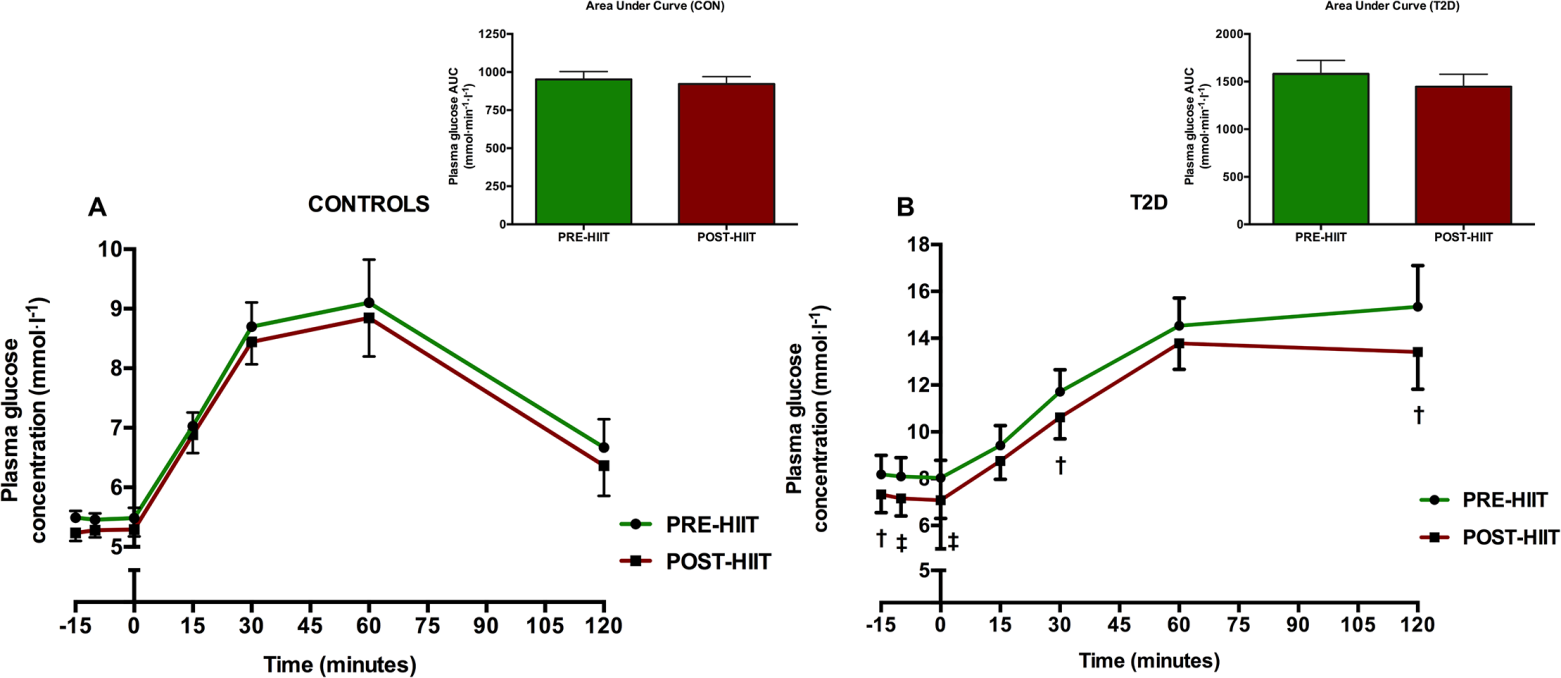
Plasma glucose levels and total AUC before and after the HIIT intervention in the CON- and T2D-group. Following 8 weeks of HIIT, the plasma glucose levels were not significantly reduced (p>0.05 at all time points) in the CON-group (A). Total AUC in the CON-group were not significantly reduced (p>0.05) (A). In the T2D-group (B), plasma glucose concentrations were significantly lowered at time points -15 min (p = 0.03 as denoted by †), -10 min (p = 0.003 as denoted by ‡), 0 min (p = 0.003 as denoted by ‡), 30 min (p = 0.03 as denoted by †) and 120 min (p = 0.03 as denoted by †). AUC was not statistically changed (p = 0.0982) (B).

**Fig 10:**
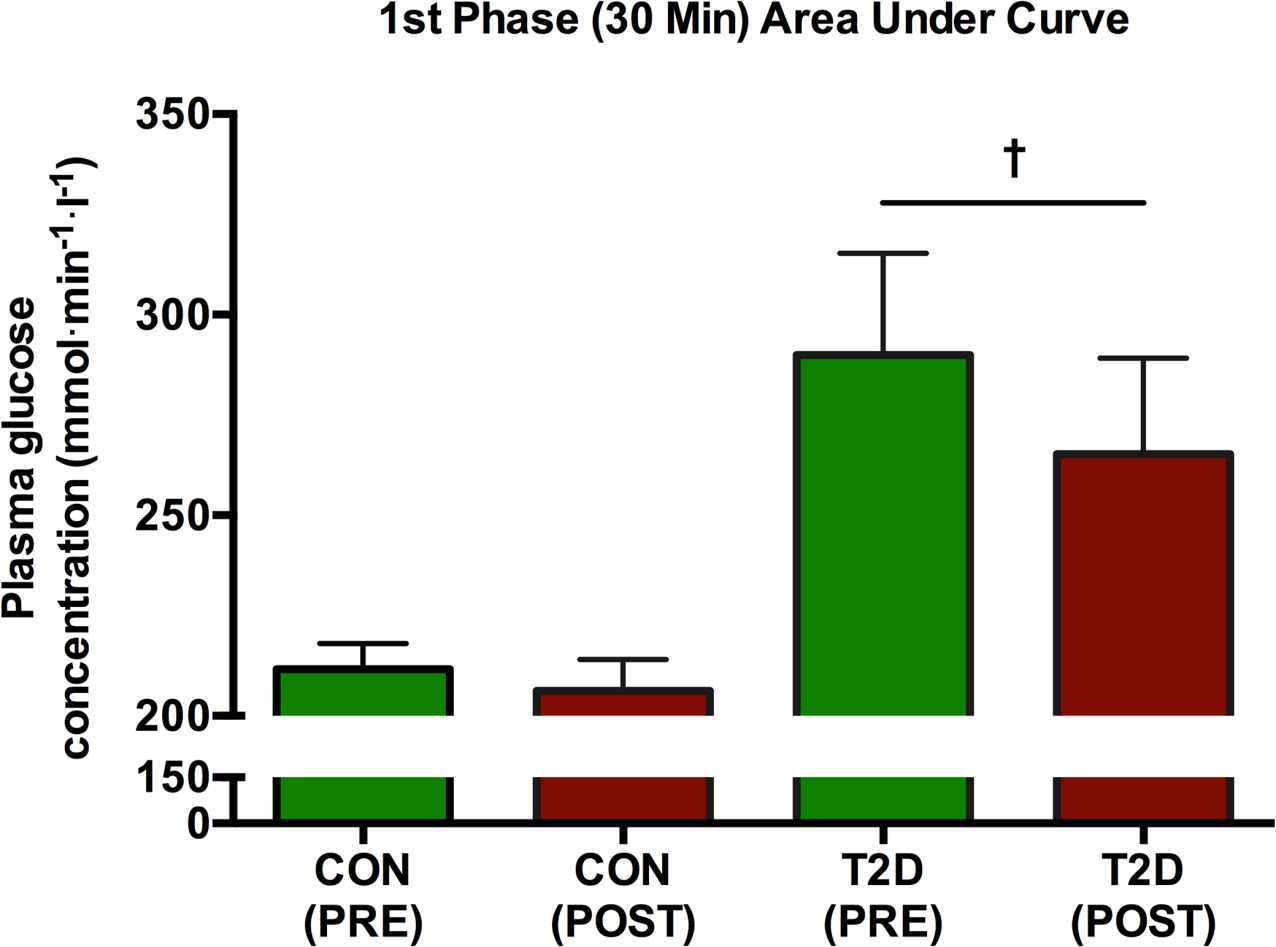
1st phase AUC after 30 minutes of the plasma glucose concentration. Following 8 weeks of HIIT, the CON-group had unaltered (p>0.05) 1st phase AUC of plasma glucose concentration, whereas the T2D-group had reduced p = 0.03 its 1st phase plasma glucose concentration (as indicated by †).

**Fig 11:**
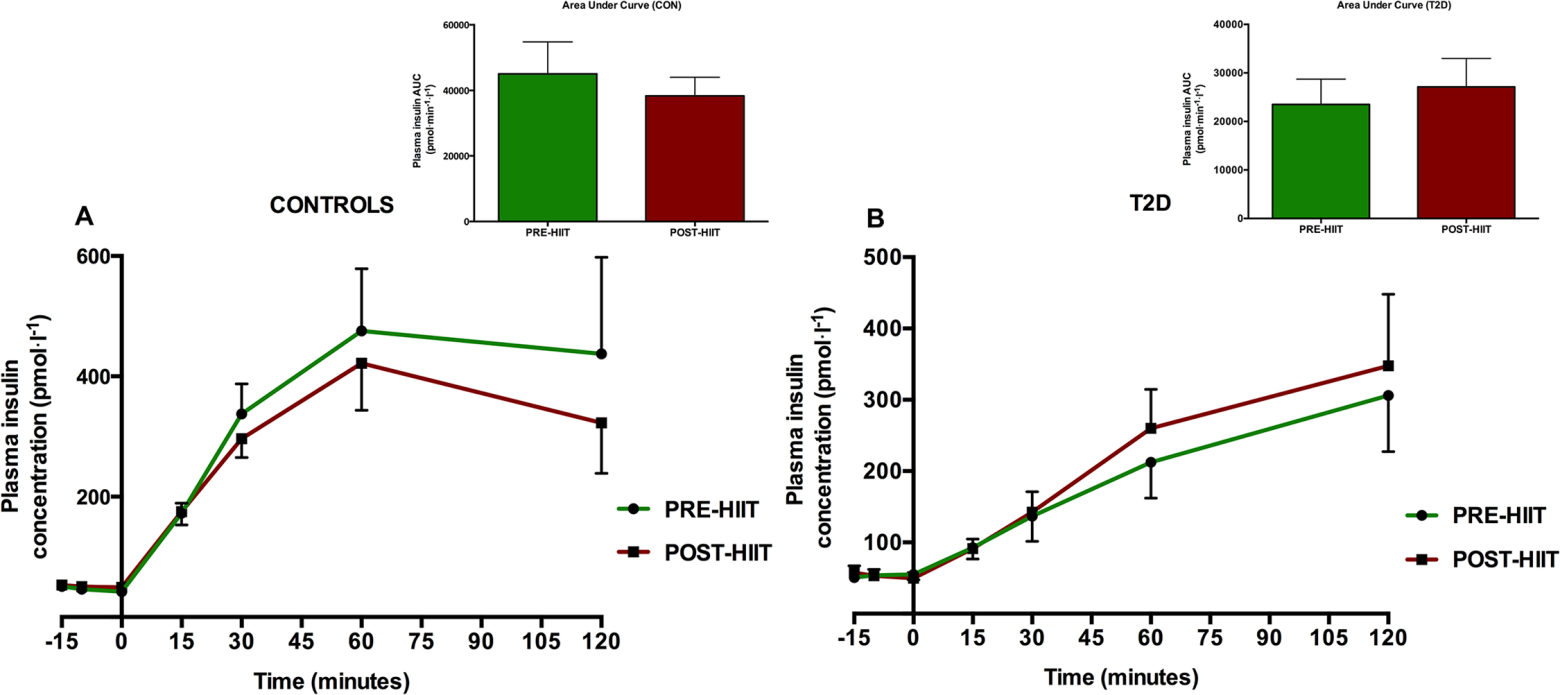
Plasma insulin levels before and after the HIIT intervention. Following 8 weeks of HIIT, the fasting insulin levels were not significantly changed in the CON-group (A) and T2D-group (B) (p>0.05 at all time points). AUC in the CON-group (A) and T2D-group (B) were not significantly reduced (p>0.05).

**Fig 12:**
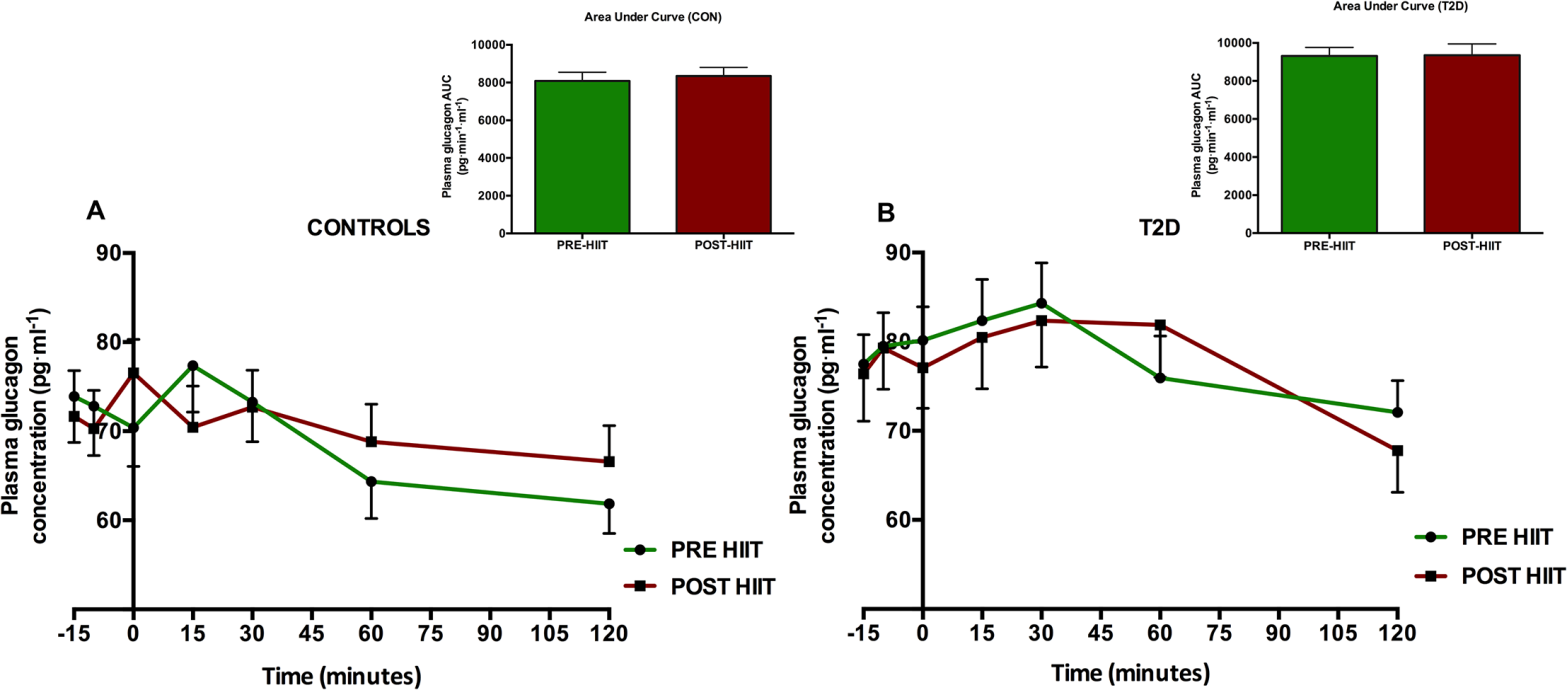
Plasma glucagon levels before and after the HIIT intervention. Following 8 weeks of HIIT, the fasting glucagon levels were not significantly changed in any group (p>0.05 at all time points). Additionally, AUC in the both groups was not significantly changed (p>0.05).

Following 8 weeks of HIIT, fasting and postprandial plasma glucose concentration was unaltered (p>0.05 at all time points) in the CON-group (Fig 9A). Also the plasma glucose AUC was unaltered (p = 0.35) after HIIT in the CON-group (Fig 9A). The T2D-group had changed its glucose significantly at time points -15 min (p = 0.03), -10 min (p = 0.003), 0 min (p = 0.003), 30 min (p = 0.03) and 120 min (p = 0.03) (Fig 9B). In the T2D-group, the AUC was 1580±142.3 mmol·min·l^-1^ at baseline and 1447±130.0 mmol·min·l^-1^ post intervention, however this did not amount to a significant change (p = 0.098) (Fig 9B). In the CON-group, 1^st^ phase plasma glucose AUC over 30 minutes was not significantly changed by the intervention effect (p = 0.36) (Fig 10). In the T2D group, however, there was a significant 1^st^ phase glucose decrease from 289.9±25.4 mmol·min·l^-1^ to 265.3±23.9 mmol·min·l^-1^ (p = 0.03) corresponding to a change of 8±3% (Fig 10). In both groups, the insulin and glucagon concentrations and their 2-hours’ AUCs were not significantly changed (p>0.05) compared to baseline (Figs 11A, 11B and 12).

### Blood pressure, exercise capacity and lipid status

Following 8 weeks of HIIT, both the CON-group and the T2D-group significantly reduced their BPs (CON: p = 0.001; T2D: p = 0.0002), BPd (CON: p = 0.0009; T2D: p<0.0001) and BPm (CON: p = 0.0002; T2D: p<0.0001) (Table 1).

**Table 1:** Baseline characteristics before and after 8 weeks of high intensity interval training. Values are means ± standard error of mean (S.E.M.).

	CON-group		T2D-group	
	Before (week 0)	After (week 9)	Before (week 0)	After (week 9)
Female/male	8/5		7/3	
Age (years)	52±2		56±2	
Systolic blood pressure (mmHg)	136.5±3.6	128.8±2.9††	134.8±2.9	123.6±2.9†††
Diastolic blood pressure (mmHg)	87.9±2.3	81.6±1.6†††	87.5±2.1	81.1±2.1†††
Mean arterial pressure (mmHg)	104.1±2.5	96.7±1.7†††	103.3±2.1	95.3±2.1†††
Total cholesterol (mmol·l^-1^)	5.26±0.18	5.05±0.15	4.21±0.45	3.83±0.47
Triglycerides (mmol·l^-1^)	1.42±0.19	1.26±0.10	1.78±0.32	1.34±0.17
HDL (mmol·l^-1^)	1.29±0.11	1.37±0.08	1.40±0.10	1.47±0.09
LDL (mmol·l^-1^)	3.32±0.21	3.12±0.19	1.99±0.36	1.76±0.40
Body weight (kg)	88.82±3.97	87.11±3.93†††	91.23±3.55	88.23±3.43††
BMI (kg·m^-2^)	30.53±0.84	29.94±0.86†††	31.14±1.24	30.09±1.18††
Lean body mass (kg)	54.77±3.83	54.52±3.61	54.13±2.57	53.22±2.62††
Body fat (%)	31.69±1.93	31.23±3.61	33.10±2.56	32.50±2.63
$\dot{\text{V}}$O_2max_ (ml·min^-1^)	2291±183.5	2410±159.5†	1963±124.0	2147±110.8†
$\dot{\text{V}}$O_2max_ relative to body weight (ml·min^-1^·kg^-1^)	25.96±1.84	27.89±1.63††	21.91±1.32	24.80±1.02††
Wattage_max_ during incremental bike test (W)	229.23±12.32	253.46±13.11†††	203.50±11.16	224.50±12.01††
Wattage_max_ during incremental bike test (W·kg^-1^)	2.61±0.13	2.93±0.12†††	2.25±0.15	2.57±0.15††
Maximal heart frequency (bpm) during $\dot{\text{V}}$O_2max_	176±2	170±3†	168±3	164±3†

† indicates significant difference within group from before HIIT. One symbol indicates p<0.05, two symbols indicate p<0.01 and three symbols indicate p<0.001.

Absolute $\dot{\text{V}}$O_2max_ changed significantly in both groups (CON: p = 0.01; T2D: p = 0.03) with a concomitant significant increase of relative $\dot{\text{V}}$O_2max_ (CON: p = 0.004; T2D: p = 0.002). Additionally, both groups increased absolute (CON: p<0.0001; T2D: p = 0.007) and relative (CON: p<0.0001; T2D: p = 0.002) maximal power output during incremental $\dot{\text{V}}$O_2max_ on the final examination (Table 1). Maximal heart rate decreased during incremental $\dot{\text{V}}$O_2max_ in both groups (CON: p = 0.01; T2D: p = 0.01) (Table 1).

No effects were demonstrated on lipid status in either group. However, in the T2D-group, post measurements of triglycerides and total cholesterol were borderline significant (p = 0.06 and 0.07) compared to baseline. HDL concentration in the CON-group was nearly significant (p = 0.06) as opposed to pre exercise values (Table 1).

### Anthropometry

**Fig 13:**
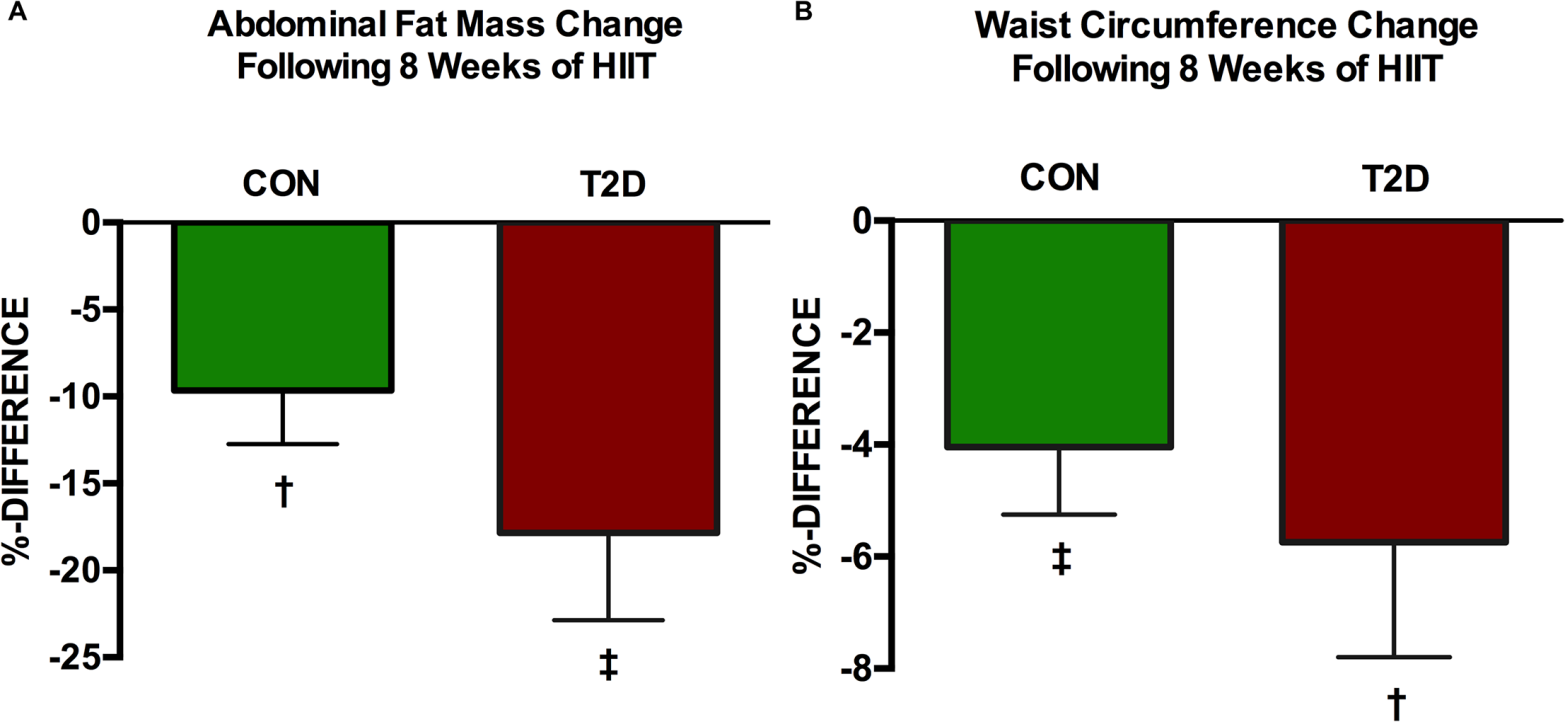
Percentage change in abdominal fat mass (A) and waist circumference (B) after 8 weeks HIIT intervention. The CON-group lowered its abdominal fat mass significantly (p = 0.02 as denoted by †) by -9.66±3.07% and the T2D-group by -17.84±5.02% (p = 0.004 as denoted by ‡). Waist circumference changed significantly in both groups by -4.04±1.20% (p = 0.006 as denoted by ‡) in the CON-group and by -5.74±2.05% (p = 0.02 as denoted by †) in the T2D-group.

Abdominal fat mass changed significantly in both groups after 8 weeks of HIIT. The CON group lowered its abdominal fat mass significantly (p = 0.02) from 3.36±0.30 kg to 3.03±0.29 kg, corresponding to percentage change of -9.66±3.07% (Fig 13A). This was also confirmed with the waist circumference measurement changing significantly from 98±3 cm to 94±3 cm (p = 0.006) (Fig 13B). Among the T2D patients, abdominal fat mass was reduced significantly (p = 0.004) from 3.06±0.28 kg to 2.57±0.32 kg. This corresponded to a percentage change of -17.84±5.02% (Fig 13A). There was also a significant reduction of the waist circumference in the T2D group from 100±2 cm to 94±3 (p = 0.02) (Fig 13B). BMI was significantly reduced in both groups, thus by p = 0.0005 in the CON-group and by p = 0.003 in the T2D-group (Table 1). Lean body mass was significantly changed (p = 0.003) in the T2D-group, whereas it remained unaltered in the CON-group (p>0.05) (Table 1). Total body fat was unaltered (p>0.05) in both groups compared to baseline (Table 1).

## Discussion

Most importantly, in this non-invasive study, we have demonstrated that 8 weeks of low volume HIIT improves glycaemic control as determined by reduced average fasting venous glucose concentration, 2-hour OGTT end point concentration and HbA_1C_ in T2D patients. Additionally, HOMA-IR and HOMA-%β were significantly improved in the T2D-group. Maybe most importantly, the DI was significantly improved. The postprandial hyperglycaemia as determined by the OGTT-values was significantly changed at 5 out of 7 time points following HIIT as well as 1^st^ phase AUC was significantly reduced. Additionally, all participants fulfilled the HIIT intervention indicating that it could be integrated as a future exercise strategy in inactive T2D- and CON-groups. Last-mentioned indicates a pivotal finding–it magnifies *de facto* HIIT as being an intriguing and seemingly optimal exercise strategy in the post-modern world in which time commitment and total exercise volume are attempted to be reduced.

### Pancreatic β-cell function

To the extent that the DI represents pancreatic β-cell function, we have demonstrated that 8 weeks of supervised low volume HIIT on cycle ergometer ameliorates pancreatic β-cell function significantly. Importantly, the DI, which is an acknowledged measure of pancreatic β-cell function adjusted for insulin sensitivity and which may predict the development of T2D, has been demonstrated to be augmented following exercise with different intensities. Karstoft and colleagues reported that interval walking (5 weekly sessions of 60 min over 4 months with 3 min intervals of fast ≥70% of $\dot{\text{V}}$O_2max_ and slow walking ~40% of $\dot{\text{V}}$O_2max_) significantly improved DI as opposed to continuous walking in adults with T2D [10], which was reflected in increased insulin sensitivity, but still an unaltered insulin secretion. In another study that was 8 months in length, treadmill and elliptical training at regular moderate intensity (40–55% of $\dot{\text{V}}$O_2peak_) showed to improve DI significantly more than vigorous intensity (65–80% of $\dot{\text{V}}$O_2peak_) in sedentary, adipose, moderately dyslipidemic participants [11]. Interestingly, high intensity resistance training over 12 weeks with 3 sessions per week in overweight sedentary young men improved the insulinogenic index and oral disposition index [30]. In our study, DI increased in the T2D group, although neither ISI_composite_ nor IGI were significantly improved. However, given the intrasubject heterogeneity in ISI_composite_ and IGI following 8 weeks of HIIT (Fig 7B) and the hyperbola fitting (Fig 8C and 8D), this could indicate that the ability of the pancreas to partially compensate for a lowered insulin resistance has been improved as opposed to baseline.

It has become clear that glycaemic control seems to be more improved by HIIT as opposed to regular moderate intensity exercise [15,16,31]. In order to assess glycaemic control, insulin secretion may be a better predictor of training responsiveness of glycaemic control as opposed to insulin sensitivity [32]. Nevertheless, equivocal findings of insulin secretion have been reported [33–35]. Although not extensively validated, the IGI assists to assess the level of insulin secretion [36]. Following regular exercise, individuals with a healthy functional pancreas normally have a lowered insulin secretion relative to an improvement of insulin sensitivity. However, in a dysfunctional pancreas where the β-cell function is impaired, exercise augments insulin secretion concomitantly with an improvement of insulin sensitivity [35,37,38]. Here, we show no effect of insulin secretion following 8 weeks of HIIT. Though a slight tendency to be decreased in the CON-group and increased in the T2D-group, the unaltered insulin secretion may be explained by several physiological phenomena: a feasible explanation to the observed low and unaltered insulin secretion could be the deteriorating effect of glucotoxicity that is present in T2D patients. Considerable research demonstrates that long-term exposure to glucose can reduce the function of pancreatic β-cells *in vivo* [39] and *in vitro* [40]. To our knowledge, the insulinogenic effect of long-term exercise (≥ 12 months) in a T2D population remains to be elucidated.

The mechanisms behind the improved pancreatic β-cell function to increase insulin action could be multifaceted, including perhaps an improved coordinated feedback loop between liver (decreased hepatic gluconeogenesis), muscle (attenuated insulin resistance) and pancreas (slowly wakening of β islets to secrete insulin). However, given that T2D is a disease characterised by perturbations in several organs, anti-inflammatory cytokines secreted by both adipocytes (e.g. adiponectin) and myocytes (e.g. IL-6) could be involved in the improvement of pancreatic β-cell function.

### Insulin sensitivity

Research of HIIT in non-healthy populations is sparse, however growing. The broad acceptance of OGTT as a clinical procedure has resulted in a growing number of research experiments attempting to explain the mechanisms behind the pathogenesis of T2D. Although others and we make use of OGTT-derived surrogate indices from which we must make careful interpretations, these indices still provide us with pivotal and detailed information that can have significant influence on the future treatment and research of T2D and especially the lives of these patients. Normally, regularly performed exercise lowers basal insulin levels and attenuates the insulin response to glucose load [41–44] suggesting that peripheral insulin sensitivity is increased.

T2D is associated with combined impaired pancreatic endocrine function and insulin sensitivity. Exercise has proven particularly beneficial as to modifying the risk of developing T2D [21]. In the T2D population, the importance of regular exercise in relation to insulin sensitivity in existing research is surprisingly equivocal. In some studies, both regular moderate intensity exercise [45–48] and HIIT [10,49,50] have shown to improve insulin sensitivity. However, few research has however demonstrated that insulin sensitivity remained unaltered following HIIT [33] and moderate intensity exercise [33,35,46]In addition, there are multiple studies finding improved insulin sensitivity among sedentary non-diabetic subjects [24,41,51]An intriguing study demonstrated that 7 consecutive days of exercise at 60–75% of heart frequency reserve improved ISI_composite_ with additional improvement of AUC in a T2D population [45]. In our study, ISI_composite_ was unchanged in both groups (Fig 6A). Exercise responsiveness of insulin sensitivity is based on multiple OGTT-derived indices potentially reflecting different information outcomes. In the absence of hypersinsulinemic euglycemic glucose clamp to measure insulin sensitivity directly, whole-body insulin sensitivity ISI_composite_ [33], peripheral insulin sensitivity as determined by the Cederholm index [43] and even hepatic insulin sensitivity [24] indices are applied to elucidate any potential changes. However, though these indices correlate nicely with the hypersinsulinemic euglycemic glucose clamp, it is nearly impossible to unequivocally conclude the actual physiological *in vivo* insulin sensitivity change. Additionally, metformin, a commonly prescribed oral hypoglycemic agent, could interact with exercise impairing insulin sensitivity outcomes [52]

HOMA-IR and HOMA-%β represent pivotal indices of tissue insulin resistance and pancreatic function. Both HOMA-IR (Fig 4) and HOMA-%β (Fig 5) were significantly improved in the T2D group after the intervention. These findings are in agreement with other findings, however the results should be interpreted carefully. In a study, HOMA was improved in T2D patients following 6 months of aerobic exercise [53]. Moreover, a single extended bout of ~200 sec. of very HIIT in sedentary overweight men demonstrated a reduced HOMA-IR [41]In a Nordic study [54], 4 months of unsupervised walking did not have any impact on HOMA-IR among T2D patients. No training induced change in insulin sensitivity was found in obese women following 6 weeks of HIIT [55], although HIIT favourably altered body composition and specific skeletal muscle oxidative capacities were improved. However, others have found that >2.5 hours of weekly endurance exercise improved the composite insulin sensitivity index among overweight sedentary adults [51], although neither exercise type, exercise intensity nor age emerge from the results. Our findings in the T2D-group demonstrate that the attenuated insulin resistance may be dependent on exercise intensity as considerable research in traditional moderate intensity training does not alter insulin resistance significantly.

### Glycaemic control: 2-hour OGTT, fasting plasma glucose, AUC and HbA_1C_

Two hours after the initiation of OGTT, glucose levels lowered significantly from 15.34±1.76 mmol·l^-1^ to 13.41±1.59 mmol·l^-1^ after 8 weeks of HIIT (Fig 3C). Additional significant findings included fasting venous blood glucose concentrations (Fig 3A) and significant lowered 1^st^ phase AUC of glucose (Fig 10) in the T2D group. Finally, the significant decrease in HbA_1C_ in the T2D-group demonstrated a clinical importance (Fig 3B). Our findings are in agreement with other studies. Eriksen and colleagues demonstrated a 7.5% decrease in the 2-hour AUC_glucose_, although the 3-hour AUC_glucose_ was unaltered [33]. In the same study AUC_insulin_ was not altered [33] and neither was it in a group of sedentary overweight adults after 2 weeks of three weekly sessions of 4–6 30 s all-out exercise bouts [42]. Moreover, a single extended bout of ~200 sec. of very HIIT in sedentary overweight men demonstrated no significant effect on [41]. Following 2 weekly sessions of 10×6 sec. sprints interspersed by 1-minute recovery between each sprints over 8 weeks, AUC_glucose_ in obese subjects was significantly reduced [56]. Among a pre-diabetic population, both a HIIT and a moderate intensity exercise session improved late-phase AUC insulin and glucose responses to an OGTT compared with a non-exercising group [57]. In our study, the tended significant increase (p = 0.087) of insulin in the T2D group may be explained by a vaguely ameliorated ability of the pancreas to waken the insulin producing β-cells for tissue action, however without a compensatory decrease of the hyperglucagonemia.

Regarding HbA_1C_, our findings are in agreement with other studies [58–60]Interestingly, the ameliorated effect of HbA_1C_ may even be favoured solely by resistance training [61]or a combination of aerobic exercise and resistance training [62,63]. However, data are not consistent as other research has found no beneficial effect of exercise to HbA_1C_ [33,64].

### Exercise capacity and blood pressure

Low volume HIIT interventions have become popular, assumingly due to its rapid physiological remodelling capabilities. Two studies have demonstrated that even as little as 3 weekly HIIT sessions over 2 weeks markedly can improve exercise capacity and tolerability in T2D populations [8,9], sedentary obese non-diabetic participants [42] and healthy participants [65–68] as well as after 4 weeks in trained athletes [69,70]Our study demonstrated noticeable HIIT-induced improvements of absolute and relative maximal oxygen consumption. Additionally, we also observed that maximal power output during the incremental $\dot{\text{V}}$O_2max_ test was augmented in both groups with a concomitant lowered maximal heart rate compared with baseline (Table 1). The lowered maximal heart rate is somewhat surprising, however it could be explained by a compensatory adaptation of central hemodynamics [71]

T2D is linked to endothelial dysfunction and hypertension [46]. We have demonstrated significant effects on overall blood pressure following 8 weeks of HIIT (Table 1), which may be of pivotal clinical relevance [72].

### CON-group

Generally, there was an overall lack of metabolic changes in the CON-group as assessed by HOMA-IR (p>0.05), HOMA-%β (p>0.05), ISI_composite_ (p>0.05), IGI (p>0.05) and DI (p>0.05) indices. Both plasma glucose and insulin concentrations were lowered following the HIIT intervention, however not significantly (p>0.05). Given the solid evidence that regular exercise *per se* improves glycaemic control through a broad variety of physiological adaptations, our findings are both astonishing and contradictory to other research results [10]. This discrepancy could be a result from the relatively short training duration, however it is a doubtful reasoning considering the tolerability and efficacy achieved in similar interventions [41,42]. Additionally, absolute (p<0.05) and relative (p<0.01) maximal pulmonary oxygen consumption were significantly enhanced. Given the non-invasive nature of this study, a careful cause, however, could be attributable to the relatively transient improvements in insulin action that gradually vanishes after ceased exercise stimulus as demonstrated in overweight sedentary men [42]. No training induced change in insulin sensitivity was found in obese women following 6 weeks of HIIT [55], although HIIT favourably altered body composition and specific skeletal muscle oxidative capacities were improved. However, others have found that >2.5 hours of weekly endurance exercise improved the composite insulin sensitivity index among overweight sedentary adults [51], although neither type, intensity nor age emerge from the results. This indicates that there may be an early metabolic remodelling mechanism that is present in the early (≤ 24 hours) post exercise phase followed by a ‘late’ (≥ 24 hours) phase that could be characterised by not being prone to same metabolic-muscular sensitivity. It could however also be explained by the fact that the CON-group may not improve its health status markedly more.

### Abdominal fat

The 8-week HIIT period lowered significantly the abdominal fat and waist circumference in both groups (Fig 13A and 13B). Noteworthy however, DXA-scans are limited in terms of distinguishing between subcutaneous and intra-abdominal fat stores. Similar findings have been demonstrated by Boudou and co-workers [73]who applied 8 weeks of combined steady state exercise (75% of $\dot{\text{V}}$O_2max_ in 45 min) twice a week with mild interval training (5×2 min of 85% of $\dot{\text{V}}$O_2max_ interspersed by 3 minutes recovery) once a week on cycle ergometer among sixteen T2D males. Here, visceral adipose tissue as measured by magnetic resonance imaging was lowered by ~44%. Whole body mass was reduced by 1.9 kg corresponding to ~2%. In another study [74] comprising of interval training (4×4 min at 90% of HR_max_ interspersed by 3 minutes recovery at 70%) twice a week for 3 months on a treadmill among fifty-four class I obese adults, abdominal fat mass reduced by ~8% as measured by DXA-scans. Same group underwent measurement of waist circumference that was reduced with 7.2 cm (~7%). Additionally, using the same exercise protocol as Boudou et al., Mourier and colleagues [13] demonstrated that abdominal fat mass determined by means of Harpender skinfold caliper was lowered by 48% among twenty-four T2D patients. Same patient group reduced its waist circumference by 1 cm (1%). Even in overweight young males, 12 weeks of HIIT consisting of 8 sec. sprint (workload ~80–90% of HR_max_) interspersed by 12 sec. recovery over 20 minutes reduced overall fat mass by 2.0 kg and abdominal fat mass by 0.14 kg [75]. From observational studies that have been comprehensively reviewed, there seems to be an anticipated inverse relationship between measures of fat mass and distribution and measures of physical exercise [76,77].

Although documentation is sparse in terms of the effect of HIIT on abdominal fat reduction among T2D populations, the magnitude of abdominal fat loss seems to be dependent on intensity. Thus, one research [17] has demonstrated that total and abdominal fat loss seems to be lowered more by HIIT than traditional continuous endurance training. Additionally, 15 weeks of 3 weekly sessions of HIIT on bicycle ergometer among diabetic obese women with polyneuropathy were found to be distinctly effective in lowering waist circumference, neuropathic pain, and hyperglycaemia as opposed to moderate intensity aerobic training [78].

The exact underlying mechanisms of the HIIT-induced abdominal fat loss are not investigated here. However, feasible explanations hereof may include excessive production of catecholamines, especially norepinephrine as opposed to continuous endurance training that typically results in relative small gains in catecholamines [79]. Particularly, the increased occurrence of β-adrenergic receptors in abdominal fat compared to subcutaneous fat indicates that HIIT may have a superior effect as opposed to continuous endurance training to drive lipolysis in abdominal fat stores [80] although greatest absolute change in fat mass in response to exercise will normally derive from subcutaneous adipose tissue [81]To our knowledge, fat oxidation experiments following a HIIT session have not been elucidated. However, given that an excessive fat oxidation occurs following HIIT, this could be explained by a broad range of factors, including the necessity of blood lactate and hydrogen ion removal for immediate glycogen resynthesis and/or elevated growth hormone levels [82,83]. Additionally, [83] an inhibition of anaerobic glycogenolysis appears, and adenosine triphosphate resynthesis is primarily derived from creatine phosphate degradation and intramuscular triacylglycerol stores. Interestingly, one study demonstrated that abdominal lipolysis in a T2D population is unaltered after exercise [84]In theory, exercise-induced abdominal fat mass reduction could be explained by the lowering of either adipocyte number and/or adipocyte size.

### Study limitations

Here, we have demonstrated that T2D patients and matched controls are able to follow a controlled study with great compliance. Retrospectively, we could have considered conducting the different durations of the HIIT interventions. Additionally, almost every participant increased his/hers self-efficacy indicating that a pre-post survey favourably could be integrated in a future study. In this study, it was a logistical challenge to train the subjects during such intensive circumstances and in relation to equipment, which limited the number of participants. In light of the findings of the present study, we wish to conduct randomized studies in the future.

## Conclusion

The study provides evidence for the health benefits of 8 weeks of HIIT in T2D patients. These effects of HIIT involved especially ameliorated glycaemic control and pancreatic β-cell function improving peripheral insulin action and reduced abdominal fat mass. In healthy subjects performing the same training, some, but not all, of the positive health effects of HIIT were observed. We observed some intrasubject heterogeneity in the exercise responsiveness in glycaemic control and pancreatic β-cell function. This indicates that more emphasis should be addressed for individualised specific needs together along with continued supervision of the oral hypoglycaemic treatment. Finally, more focus should be addressed on even more long-term HIIT interventions and/or the seemingly attenuated hyperglycaemic effects of resistance training.

## Supporting Information

S1 CONSORT Checklist CONSORT checklist. (PDF)

S1 Protocol Trial protocol from the Ethical Committee translated. (PDF)

S2 Protocol Trial protocol to the Ethical Committee in Danish. (DOCX)
