# Supplementary material for: High Intensity Interval Training Improves Glycaemic Control and Pancreatic β Cell Function of Type 2 Diabetes Patients
Source: PLoS One. 2015 Aug 10;10(8):e0133286. doi: 10.1371/journal.pone.0133286 (PMC4530878; doi:10.1371/journal.pone.0133286)
Supplement: S1 Protocol — (PDF) [file pone.0133286.s002.pdf]

Project description – Ethic committee translated to English

Title and name: Cand. scient. (physiology), PhD-student Søren Møller Madsen
Project title: The health beneficial effects of high intensity interval training to type 2 diabetes patient

1. Background:

According to the Danish National Diabetes Register, approximately 245.000 individuals are diagnosed with type 2 diabetes (T2D), while it is estimated that equally many have T2D without knowing it. The incidence of T2D was approximately 23.000 in 2009 and approximately 750.000 individuals have incipient symptoms of T2D, also known as pre-diabetes (Sundhedstyrelsen). This is expected to increase explosively with up to 439 million in 2030 (1), and thus T2D may be regarded as one of the greatest global health care challenges of the 21st century.

The onset of T2D is caused by a combination of peripheral insulin resistance and β-cell dysfunction, which induce a lower rate of glucose uptake into skeletal muscle, compared to non-diabetic individuals. Genetic predisposition, obesity and physical inactivity are three important causes of these metabolic perturbations. Additionally, it has been suggested that T2D also may be the result of impaired β-cell regeneration and decreased β-cell mass in the pancreas (2-6). Concurrent with the development of T2D, intrinsic metabolic perturbations caused by the disease may cause complications, including vascular diseases such as e.g. hypertension, acute myocardial infarction, intermittent claudication and microangiopathy (7).

In addition to the above-mentioned T2D characteristics, the disease is believed to cause changed blood flow patterns in the muscle tissue. In one study, it is demonstrated that the physiological morphology of capillaries among T2D patients with and without micro angiopathy is disturbed, followed by decreased capillary blood volume and blood flow (7), which can be induced by increased endothelial dysfunction, and thus a reduced capability of the vessels to vasodilate. Endothelial dysfunction is regarded as a critical early event in the development of atherosclerosis prior to great morphological changes and clinical symptoms.

It is well-known that the blood flow in the skeletal musculature increases proportionally with increasing oxygen demand (8, 9). In a recently published study (9), it is shown that hypertensive patients have a lower blood flow during one-legged knee-extensor exercise compared with normotensive subjects. Among other things, T2D is associated with endothelial dysfunction, but currently it is not known whether a changed blood flow is seen in this patient group. The same research group also demonstrated that 8 weeks of high intensity interval training (HIIT) reduces blood pressure in the leg (measured in a. femoralis) in hypertensive patients followed by lower conductance levels. Whether the blood flow – by using flow-mediated vasodilation (FMD) – is changed in patients with T2D following 8 weeks of HIIT is not known, but has been examined in individuals with coronary disease, in which FMD was immediately improved following *one* time of HIIT (10).

Exercise has proven particularly beneficial as to modifying the risk of developing T2D (11). However, there is no agreement on the nature of specific exercise therapy necessary to provide such health benefits, particularly with regard to the volume-intensity relationship.

HIIT is an exercise strategy that involves short strenuous bouts, each individually followed by a recovery period that is adequately long enough to recover in a view to performing at maximal effort subsequently (12-14). In several experiments, researchers have used HIIT in order to establish an important metabolic paradigm: HIIT improves the maximal activities of mitochondrial enzymes as well as HIIT is associated with reduced glycogen utilisation and lactate accumulation during matched-work exercise (10,13,15-16). Moreover, HIIT increases performance during tasks that predominantly

are based on aerobic metabolism (13,15). Importantly also, HIIT proves highly health beneficial for insulin sensitivity and glucose homeostasis in sedentary individuals (17).
For these reasons, HIIT strategy seems to be a good choice for both healthy and especially diseased patients with T2D. Additionally, it has proven a time-efficient strategy, thus potentially more individuals are able to comply with these exercise regimes compared to time-consuming strategies such as both endurance and resistance training (14, 17). In fact, some research groups have demonstrated only 1/10 time spent on HIIT compared to conventional endurance training, which for a long time have been the most preferable exercise strategy for both athletes and patients with T2D (14; 17). Ultimately, since many patients with T2D drop out of these programs, HIIT seems also to be an important strategy as regards motivation and adherence (15).
Direct catherisations and thermodilution in artery and vein in the femoral region are believed to be the most accurate methods to measure *in vivo* hemodynamics in humans. The *non-invasive* inherent characteristics of an ultrasound system allows one to assess *in vivo* hemodynamics (blood flow and diameter of vessel) in humans (18). By applying a blood pressure cuff (FMD), one can assess the vasodilatory function on skeletal muscle blood flow of major vessels (18). FMD is believed to reflect the endothelial-dependent <u>and</u> predominantly nitricoxid (NO)-mediated artery function and has been applied as a substitute for the cardiovascular function. NO is significant in terms of the regulation of systemic and peripheral blood pressure in humans, and is a potent vasodilator, which relaxes the vascular smooth muscle tissue for immediate vasodilation. By applying FMD, the method is used to isolate NO, thus the FMD-response is an index for the NO bioavailability, finally looking upon the ability to vasodilate. Hence, the method is believed to be excellent compared to other comprehensive invasive measurements (direct catherisations in artery and vein in the femoral region), which also is described earlier (18).

2. Hypotheses:

- Following 8 weeks of controlled HIIT, the T2D patients will have improved their ability to oxygenate their muscle tissue significantly, including e.g. improved vasodilation (measured by applying FMD).
- Following 8 weeks of controlled HIIT, it is expected that the glucose homeostasis and insulin sensitivity among T2D patients and healthy subjects have been improved.
- Following 8 weeks of controlled HIIT, it is expected that HbA_{1C}, the inflammatory status and lipid profile among T2D patients and healthy subjects have been improved.
- The above-mentioned hypotheses will indicate that the T2D patients – prior to the HIIT study – have a disturbed vessel-physiological morphology, which induces poor oxygen transfer to the muscle tissue.
- Following 8 weeks of controlled HIIT, it is expected that the systemic blood pressure is decreased.
- NO vasodilatory system is expected to be improved following 8 weeks of HIIT.

3. Materials and methodology:

12-lead electrocardiography (ECG)
We apply 12-lead ECG to obtain the subject's electrical activity of the myocardium. If any perturbations during the heart cycle (both before and after the initial VO_{2peak} test), he/she will be precluded from the study. The procedure is conducted by trained staff.

Dual-emission X-ray absorptiometry (DXA) scans:

DXA scans has proven a particularly accurate method to measure fat free mass, fat mass and body weight, for which reason it is a good method to evaluate any potential changes in body compositions (19).

VO_{2peak}, time trials and high intensity interval training (HIIT):

For the HIIT experiment, a Monark cycle ergometer is used. The test starts at 30 W for 1 min followed by a load that is increased incrementally at a rate of 20 W every minute until the subject can not sustain a cadence greater than 60. VO_{2peak} will be recorded as the highest VO_2 reading recorded for 20 s during the test (using an online breath-by-breath analyser Oxycon-Pro, Viasys, Hoechberg, Germany). Peak power output is calculated by averaging the power output for the final minute of the VO_{2peak} test. Additionally, ventilation volume (V_E), oxygen consumption (VO_2) and carbon dioxide production (VCO_2) will be measured.

Two similar self-paced cycling time trials (250kJ, corresponding to 10km) are performed of which the faster one will be used for later comparison (Gibala et al., 2006). First time trial will be used for familiarisation. No physiological, temporal or verbal feedback will be given, only with the exception of distance covered.

Each HIIT comprises 4-6×30-sec. cycle sprints against a resistance equivalent to 7.5% of body mass – a so-called Wingate test), with 4 min. recovery between each sprint and with 3 sessions every week (Babraj et al., 2009; Gibala et al., 2006; Burgomaster et al., 2005). 4 weeks of HIIT will be performed in both studies 2 and 3.

Oral glucose tolerance test (OGTT):

All subjects will refrain from performing any strenuous exercise for 2 days prior to the OGTT as well as the subject was instructed to fast (water is allowed) overnight while smoking and eating prior to the OGTT is not allowed. An oral glucose tolerance test (OGTT) is a 2 hours medical test, in which 75 g glucose dissolved in 100 ml of water (ordered at the local hospital pharmacy at Aarhus University Hospital and is stored in a refrigerator until application) is given followed by venous blood samples in a view to determining how fast the glucose is cleared from the blood (source). In both studies, venous blood samples will be collected at -15, -10, zero time (baseline), 15, 30, 60 and 120 minutes after the oral intake of the glucose dissolving. Centrifugation is used to separate plasma from the red blood cells and will be stored at -20°C until analysis of glucose, glucagon and insulin (metabolic parameters), lipids (triglycerides, HDL, LDL and total cholesterol) as well as interleukins and cytokines (inflammatory markers such as TNF-α, IL-6, IL-10 and c-reactive peptide).

Finally, a second OGTT will be performed after completion of the HIIT study program. All subjects will be tested either two or three days after the last bout of exercise (i.e. day 44 or 45) in order to preclude acute effects attributable to the last exercise bout.

Importantly, in order to make sure that all individuals are capable of conducting exercise, we make sure that leukocytes, HbA_{1C}, erythrocytes, thrombocytes, ASAT, ALAT, coagulation factors (II, VII and X), albumin, INR, CRP, creatinine and TSH are within normal ranges – if not, the subject will be excluded from the study.

Measurements of capillary blood glucose concentrations:

The patient will have an apparatus (Accutrend) delivered for quantifying capillary blood glucose concentration, which is determined every morning immediately after wake-up (postprandial phase) and before breakfast is consumed. For the apparatus, strips and finger dots will be given. Data will be registered in a document, on which the patient writes in two measured blood glucose concentrations on the mornings after the HIIT-interventions. Prior to using the apparatus, the patient is carefully instructed how to use the system, thus he/she will be able to conducted it for the next 8 weeks.

Effect parametres:

- Measurement of resting vasodilation before and after HIIT by applying FMD.
- Metabolism, oximetry and inflammation differences
- HIIT as a training strategy

Flow-mediated vasodilation (FMD) by applying ultrasound
Earlier in this description, FMD is explained. Below a short description of the experimental setup is explained:

- Baseline diameter of a. popliteal is examined prior to blood pressure cuff is activated (baseline). Measurement in 1 minute.
- The blood pressure cuff is pumped up manually for 5 minutes (reactive hyperaemia FMD/shear stress) to a pressure ≥ 200 mmHg (suprasystolic pressure) after which the pressure is dropped to 0 mmHg. In this phase, the blood flow (ml min^{-1}) of a. popliteal within 15 seconds and the diameter of a. popliteal at 45, 90, 180 and 300 seconds.
- The measurement of active hyperaemia is conducted ≥ 5 minutes.

Statistical analysis:
During the statistical evaluation of the data, the average ± S.E.M will be presented. Data from the OGTT (before and after HIIT) will be analysed by applying *two-way reapeated measures ANOVA* with *post hoc* Student Newman-Keuls tests. Differences between before and after VO_{2peak} and blow flow characteristics will be analysed by applying *Student's paired t-test. Pearson's correlation coefficient* will be applied in order to evaluate bivariate correlations between baseline values and changes of the following variables: metabolic markers, oximetry, inflammation, cytokines, blood flow (FMD) as well as VO_{2peak} in each experiment. P-values below 0.05 will be assumed as statistically significant. The statistical programmes STATA (for measurement) and Prism GraphPad (for visual representation) will be applied.
The Lilliefors test will be applied to test whether data is normally distributed. For data not fulfilling this criterion, a Wilcoxon signed rank test will be used. STATA (for measurement) and Prism GraphPad (for visual version) are applied.

Study population and recruitment:
17 patients with T2D and 17 control subjects will be attempted to be recruited for the studies). However, only 10 healthy subjects are needed for study 1. All will recruited from Sygehus Vendsyssel (Hjørring). All participants are given both oral and written information about the purpose, nature and potential risks before they gave a written informed consent to participate in the study. Clinical experiments carried out in humans will occur in agreement with the local Human subject Ethics Committee of Region Midtjylland. Additionally, all procedures conform to the declaration of Helsinki (**World Medical Association**).

Additionally, we will look into the possibility of making a methodology study on the basis of all the data collected from 2011-14. Here, a short theoretical description will be given:

Contrast enhanced ultrasound (CEUS) specificity:
IU22 Philips ultrasound scanner together with a micro bubble agent (SonoVue® and Optison) will be applied with the linear transducer L9-3 for the contrast scan. Harmonic power-Doppler imaging is performed at a transmission frequency of 3 MHz with a linear-array transducer at a mechanical index of 0,07 (Madsen & Østergaard, unpublished data). From unpublished data, it is found that individuals weighing more than 60 kg, a bolus of 0.3 ml of SonoVue® is needed. A bolus of 0.15 ml SonoVue® is

needed when weighing less than 60 kg (Madsen & Østergaard, unpublished data). A cannula is inserted in the non-dominant arm. For standardisation, the ROI of each subject will be drawn with a pen on the skin in a 25% distance from elbow joint to hand joint or from hip joint to knee joint. Additionally, each subject will meet fasting from the night before. All subjects will also refrain from performing any strenuous exercise for 2 days prior to the experiment.

4. Research plan (August 2014-December 2015):

In the time of writing, I will predominantly handle data from the diabetes study obtained from the study conducted between April and June at Hjørring Sygehus. From this, one or perhaps two articles may be published. Additionally, I will look into both doing an exercise-diabetes review and a methodology study (obtained from all contrast-enhanced ultrasound studies from 2011-2014). Additionally, I will spend 4 months at the Norweigian School of Sport Sciences (NIH) to exchange and develop new research experiences under close guidance from professor Jørgen Jensen, who conducts research within exercise, glucose metabolism and intracellular signalling of skeletal muscles. From this stay, it may be possible to be co-writer of ongoing studies. Finally, by now there may be time to conduct research for both my supervisor associate professor Per Bendix Jeppesen and my co-supervisor associate professor Kristian Overgaard, who research in arthritis and HIIT interventions respectively.

Additionally, I will be part of an interdisciplinary research environment, in which there will be great possibilities to write articles in close cooperation with other scientists, including my supervisor Associate Professor Per Bendix Jeppesen, co-supervisor Associate Professor Kristian Overgaard and fellow PhD-students. Both positive and negative results will be published in international journals. Additionally, same working group will assist me during the experiments.

Obviously, this physiological approach along with our hypotheses may establish further importance of the metabolic role of the blood flow patterns of how to approach T2D more efficiently. Therefore, collaboration between NIH and our department seems to be of mutual benefit.

5. Perspectives:

It is the purpose of this PhD project to elucidate the role of macro blood flow patterns and metabolic changes in healthy and diabetes mellitus type 2 patients in response to high intensity interval training, and whether it is coupled to endothelial dysfunction. By the proposed experiments, we hope to achieve a more complete understanding of the linkage between macro blood flow patterns (the ability to vasodilate before and after HIIT intervention), glucose homeostasis and inflammation under exercise stimulus in T2D patients. This, in turn, will likely allow us to answer key pathophysiological and pharmacological questions in relation to exercise in general, and T2D in particular.

6. References:

(1) Shaw JE, Sicree RA, Zimmet PZ. Global estimates of the prevalence of diabetes for 2010 and 2030. Diabetes Res Clin Pract 2010 Jan;87(1):4-14.

(2) Dor Y, Brown J, Martinez OI, Melton DA. Adult pancreatic beta-cells are formed by self-duplication rather than stem-cell differentiation. Nature 2004 May 6;429(6987):41-46.

(3) Georgia S, Bhushan A. Beta cell replication is the primary mechanism for maintaining postnatal beta cell mass. J Clin Invest 2004 Oct;114(7):963-968.

(4) Jessen N, Goodyear LJ. Contraction signaling to glucose transport in skeletal muscle. J Appl Physiol 2005 Jul;99(1):330-337.

(5) Porte D,Jr, Kahn SE. Beta-Cell Dysfunction and Failure in Type 2 Diabetes: Potential Mechanisms. Diabetes 2001 Feb;50 Suppl 1:S160-3.

(6) Rhodes CJ. Type 2 diabetes-a matter of beta-cell life and death? Science 2005 Jan 21;307(5708):380-384.

(7) Womack L, Peters D, Barrett EJ, Kaul S, Price W, Lindner JR. Abnormal skeletal muscle capillary recruitment during exercise in patients with type 2 diabetes mellitus and microvascular complications. J Am Coll Cardiol 2009 Jun 9;53(23):2175-2183.

(8) Andersen P, Saltin B. Maximal perfusion of skeletal muscle in man. J Physiol 1985 Sep;366:233-249.

(9) Nyberg M, Jensen LG, Thaning P, Hellsten Y, Mortensen SP. Role of nitric oxide and prostanoids in the regulation of leg blood flow and blood pressure in humans with essential hypertension: effect of high-intensity aerobic training. J Physiol 2012 Mar 15;590(Pt 6):1481-1494.

(10) Harmer AR, McKenna MJ, Sutton JR, Snow RJ, Ruell PA, Booth J, et al. Skeletal muscle metabolic and ionic adaptations during intense exercise following sprint training in humans. J Appl Physiol 2000 Nov;89(5):1793-1803.

(11) Pedersen, B. K. & Saltin, B.; Evidence for prescribing exercise as therapy in chronic disease; Scand J Med Sci Sports, 16(Suppl 1): 3-63, 2008

(12) Gibala MJ, McGee SL. Metabolic adaptations to short-term high-intensity interval training: a little pain for a lot of gain? Exerc Sport Sci Rev 2008 Apr;36(2):58-63.

(13) Burgomaster KA, Hughes SC, Heigenhauser GJ, Bradwell SN, Gibala MJ. Six sessions of sprint interval training increases muscle oxidative potential and cycle endurance capacity in humans. J Appl Physiol 2005 Jun;98(6):1985-1990.

(14) Gibala MJ, Little JP, van Essen M, Wilkin GP, Burgomaster KA, Safdar A, et al. Short-term sprint interval versus traditional endurance training: similar initial adaptations in human skeletal muscle and exercise performance. J Physiol 2006 Sep 15;575(Pt 3):901-911.

(15) Burgomaster KA, Heigenhauser GJ, Gibala MJ. Effect of short-term sprint interval training on human skeletal muscle carbohydrate metabolism during exercise and time-trial performance. J Appl Physiol 2006 Jun;100(6):2041-2047.

(16) Saltin B, Nazar K, Costill DL, Stein E, Jansson E, Essen B, et al. The nature of the training response; peripheral and central adaptations of one-legged exercise. Acta Physiol Scand 1976 Mar;96(3):289-305.

(17) Babraj JA, Vollaard NB, Keast C, Guppy FM, Cottrell G, Timmons JA. Extremely short duration high intensity interval training substantially improves insulin action in young healthy males. BMC Endocr Disord 2009 Jan 28;9:3.

(18) Thijssen DH, Black MA, Pyke KE, Padilla J, Atkinson G, Harris RA, et al. Assessment of flow-mediated dilation in humans: a methodological and physiological guideline. Am J Physiol Heart Circ Physiol 2011 Jan;300(1):H2-12.

(19) Owens S, Gutin B, Allison J, Riggs S, Ferguson M, Litaker M, et al. Effect of physical training on total and visceral fat in obese children. Med Sci Sports Exerc 1999 Jan;31(1):143-148.
