# Supplementary material for: High Intensity Interval Training Improves Glycaemic Control and Pancreatic β Cell Function of Type 2 Diabetes Patients
Source: PLoS One. 2015 Aug 10;10(8):e0133286. doi: 10.1371/journal.pone.0133286 (PMC4530878; doi:10.1371/journal.pone.0133286)
Supplement: S2 Protocol — (DOCX) [file pone.0133286.s003.docx]

**Undersøgelse af blodgennemstrømningen og glukosemetabolismen i forlårsmuskulaturen hos patienter med type 2 diabetes og matchede kontrolpersoner – effekten af 8 ugers kontrolleret høj intens intervaltræning**

**Forsøgsprotokol**

Anmeldelsesnummer: 36921

## *Investigator*

Søren Møller Madsen, cand.scient. (humanfysiologi), Ph.D.-studerende

Medicinsk Endokrinologisk Afdeling, Aarhus Universitet

Aarhus Universitetshospital

Tage Hansens Gade 2

8000 Aarhus C

Telefon: 61 67 17 91

E-mail: [smmadsen@cfin.dk](mailto:smmadsen@cfin.dk)

## *Forsøgsansvarlige*

Per Bendix Jeppesen

Medicinsk Endokrinologisk Afdeling, Aarhus Universitet

Aarhus Universitetshospital

Tage Hansens Gade 2

8000 Aarhus C.

Telefon: 28 15 18 77

E-mail: [per.bendix.jeppesen@ki.au.dk](mailto:per.bendix.jeppesen@ki.au.dk)

***Interne samarbejdspartnere***

Anne Cathrine Thorup

Medicinsk Endokrinologisk Afdeling, Aarhus Universitet

Aarhus Universitetshospital

Tage Hansens Gade 2

8000 Aarhus C.

Telefon: 30 62 27 93

E-mail: [anne.cathrine.thorup@clin.au.dk](mailto:anne.cathrine.thorup@clin.au.dk)

Kristian Overgaard

Sektion for Idræt, Institut for Folkesundhed

Aarhus Universitet

Dalgas Avenue 4

8000 Aarhus C.

Telefon: 51 66 65 48

E-mail: [ko@sport.au.dk](mailto:ko@sport.au.dk)

Mads Münster Karlsson, M.D.

Sygehus Vendsyssel

Bispensgade 37

9800 Hjørring

E-mail: [mads.karlsson@dadlnet.dk](mailto:mads.karlsson@dadlnet.dk)

**Forsøgsprotokol**

**Formål**

Det overordnede formål med nærværende studie er at undersøge en mulig sammenhæng mellem blodgennemstrømningen og glukosemetabolismen i m. quadriceps før og efter 8 uger med høj intens intervaltræning (HIIT) på cykelergometer hos patienter med type 2 diabetes (T2D) og matchede kontrolpersoner.

**Baggrund og hypotese**

Type 2 diabetes (T2D) er blandt verdens hurtigst voksende livsstilsygdomme. Alene i Danmark er der ifølge Sundhedsstyrelsen ca. 245.000 diagnosticerede T2D-patienter. Hertil kommer, at det er estimeret, at lige så mange menes at have T2D uden at have kendskab til det. Derudover har op til 750.000 danskere begyndende diabetiske komplikationer, såkaldt prædiabetes (31). T2D udgør også en sundhedsmæssig risiko i resten af verden; i 2010 mentes 285 millioner at have T2D, og dette forventes at øge eksplosivt med op til 439 millioner i 2030(1). Dermed fremstår T2D som en af det 21. århundredes største globale sundhedsudfordringer.

T2D skyldes en kombination af perifer insulinresistens og β-celle dysfunktion, hvilket især giver anledning til lavere glukoseoptag (dvs., at GLUT4 translokationsmekanismen er forringet) i skeletmuskulaturen sammenlignet med ikke-diabetiske individer. Genetisk disposition, fedme og fysisk inaktivitet er tre vigtige faktorer, der ligger til grund for disse metaboliske forstyrrelser. Det menes også, at T2D kan opstå som følge af en β-cellernes manglende evne til at regenerere samt nedsat β-celle masse i bugspytkirtlen(2-6). Samtidig med udviklingen af T2D, kan de nævnte metaboliske forstyrrelser også give anledning til at udvikle en række vaskulære sygdomme, herunder f.eks. hypertension, myokardieinfarkt, claudicatio intermittens og mikroangiopati(7).

Foruden ovenstående T2D karakteristika, menes sygdommen også at give anledning til ændret blodgennemstrømning i muskelvævet. I et studie er det påvist, at den kapillærfysiologiske morfologi hos T2D patienter med og uden mikroangiopati er forstyrret, med især nedsat kapillær blodvolumen og blodgennemstrømning til følge(7), hvilket kan være fremkaldt af stigende endothelial dysfunktion, og dermed nedsat evne i karrene til at vasodilatere. Endothelial dysfunktion betragtes som en kritisk tidlig begivenhed i udviklingen af aterosklerose, som *går forud for* store morfologiske ændringer og kliniske symptomer. Vurderingen af flowmedieret vasodilatation (FMD) er en *non-invasiv* procedure ved brug af klassisk ultralydsskanning og en blodtryksmanchet med henblik på vurdere den vasodilatoriske funktion *in vivo* (8). FMD menes at reflektere den endothelial-afhængige og overvejende nitridoxid (NO)-medieret arteriefunktion og har været brugt som surrogat for den kardiovaskulære funktion. NO er afgørende for reguleringen af både systemisk og perifer blodtryk hos mennesker, og optræder som en potent vasodilator, der relakserer det vaskulærer glatte muskelvæv mhp. vasodilatation. Ved brug af FMD forsøges NO at blive isoleret, således at FMD-svaret er et indeks for NO-biotilgængeligheden og dermed evnen til at kunne vasodilatere. Således menes metoden også at være ganske fremragende i forhold til omfattende invasive målinger (direkte katerisationer i hhv. arterie og vener i femoralregionen, som tidligere ansøgt), hvilket også er beskrevet tidligere (8).

Det er velkendt, at blodgennemstrømningen i skeletmuskulaturen stiger i takt med et stigende iltkrav (9,10). I et for nylig publiceret studie (10) er det blevet vist, at hypertensive patienter har lavere blodgennemstrømning under et-bens knæekstensor arbejde sammenlignet med normotensive forsøgspersoner. T2D er bl.a. forbundet med endothelial dysfunktion, men det vides ikke præcist om det giver anledning til ændret blodgennemstrømning hos denne patientgruppe. Samme forskningsgruppe viste også, at 8 ugers høj intens træning sænker blodtrykket i benet hos hypertensive patienter med dertilhørende lavere konduktans. Hvorvidt blodgennemstrømningen (ved brug af FMD) ændres hos T2D patienter efter 8 ugers høj intens træning vides ikke, men er bl.a. blevet undersøgt hos individer med koronarsygdom, hvor FMD blev øjeblikkelig forbedret efter høj intens træning én gang (11).

Der er ikke enighed om, hvilken specifik træningsstrategi, der er nødvendig for at tilvejebringe ovenstående helbredsfordele for T2D patienter; i særdeleshed med hensyn til forholdet mellem volumen og intensitet. Høj intens intervaltræning (HIIT) er en træningsstrategi, som omfatter korte anstrengende omgange, hver individuelt efterfulgt af en restitutionsperiode, der er tilstrækkelig lang til, at forsøgspersonen er i stand til at komme sig med henblik på at kunne udføre tilsvarende maksimale anstrengelse efterfølgende – i de nævnte referencer udført på en Wingate cykel, hvor der sprintes maksimalt med en vægt svarende til 7.5% af ens kropsmasse over 30 sekunder med efterfølgende 3-4 minutters pause, hvorpå det gentages 3-5 gange (12-14). I adskillige forsøg har forskere brugt HIIT for at etablere et afgørende metabolisk paradigme: HIIT forbedrer den maksimale aktivitet af mitokondrielle enzymer såvel som HIIT er forbundet med reduceret glykogenforbrug og laktatakkumulation under arbejdsreguleret fysisk aktivitet (14-17). Dertil kommer, at HIIT øger præstation under arbejde, som overvejende er baseret på den aerobe metabolisme (14,15). Ydermere er det påvist, at HIIT er særlig gunstig for insulinsensitiviteten og glukosehomøostasen hos stillesiddende individer (18). Trods relativ stort videnskabeligt belæg for HIIT’s helbredsfordele indebærer Wingate protokollen potentielt op til to kritiske problemer for T2D patienter: 1) testen *per se* kræver høj motivation, og den ekstremt krævende træningsform (maksimal udfoldelse hver gang) kan inducere svær træthed, og 2) testen kræver specialiseret ergometer (jf. Wingate ergometer) og kan forekomme upraktisk at udføre både i kliniske og hjemlige sammenhænge. Derfor er det blevet forsøgt at udvikle en alternativ HIIT protokol, som omfatter en relativ mildere belastning for denne patientgruppe og med tanke om at kunne gennemføre den. Den alternative HIIT protokol omfatter 10×60 sekunders intervaller afbrudt af 60 sekunders pause mellem hvert interval, og med en arbejdsintensitet svarende til en kraft, der under en VO_2peak_-test fremkalder ~90% af den maksimale hjertefrekvens. Så vidt vides er der to forskningsgrupper, som har testet denne træningsform på henholdsvis T2D patienter (19) og stillesiddende individer (20) begge udført over to uger med signifikante metaboliske effekter. Dette kan indikere, at denne alternative HIIT træningsstrategi kan inducere væsentlige metaboliske effekter, herunder reducere den diabetiske hyperglykæmi og dermed give anledning til bedre helbred for denne patientgruppe.

Formålet med dette studie er således at øge vores viden om, hvordan FMD omkring m. quadriceps hos patienter med T2D og matchede kontrolpersoner før og efter 8 uger langt HIIT-forløb for på sigt at kunne bruge denne viden til at forstå disse forsøgspersoners karfysiologi i skeletmuskulaturen – og forhåbentlig forbedre behandlingen.

Hypoteser:

1. Efter 8 ugers kontrolleret HIIT vil T2D patienterne have forbedret deres evne til at oxygenere muskelvævet markant, bl.a. som følge af bedre vasodilatation (målt ved brug af FMD).
2. Efter 8 ugers kontrolleret HIIT forventes det, at T2D patienterne har forbedret glukosehomøostase og insulinsensitivitet.
3. Ovenstående 2 hypoteser vil indikere, at patienterne – forud for HIIT-studiet – har forstyrret karfysiologisk morfologi, hvilket betinger dårligere iltoverførsel til muskelvævet.
4. Efter 8 ugers kontrolleret HIIT forventes det, at det overordnede systemiske blodtryk vil falde.

**Antal af forsøgspersoner**

På nuværende tidspunkt forelægger der ikke tilstrækkelig normalmateriale hos patientkategorien til at give mulige standardafvigelser (SD) eller δ (ændringer af endothelial dysfunktion) i forhold til antallet af patienter, der skal inkluderes i projektet. Dog er det forsøgt med figur 1 nedenfor (styrke=80% og signifikansniveau, α=5%) at illustrere, hvor stort et antal af patienter, der behøves ud fra forskellige SD og δ. I vores studie sættes antallet af patienter til 34 (17 T2D patienter og 17 matchede kontrolpersoner), idet der forventes drop-out på 20%, styrke på 80%, SD på 4 og δ på 2. Antallet af forsøgspersoner forventes at være tilstrækkelig, idet andre studier (9,10) har inkluderet henholdsvis 8 og 7 T2D patienter som forsøgspersoner. Ligeledes har der i et tredje lignende studie med fokus på HIITs effekt på hypertensive patienter blevet inkluderet i alt 21 forsøgspersoner (10).


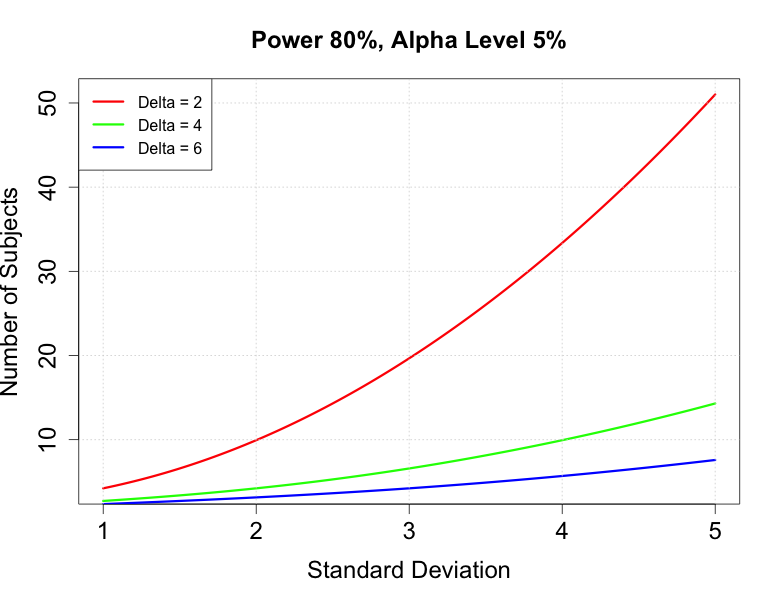


**Figur 1:** viser antallet af forsøgspersoner med en fastlagt styrke på 80% og signifikansniveau på 5% samt forskellige SD og δ (ændringer af endothelial dysfunktion). De stiplede linjers skæringspunkt illustrerer det forventede forhold mellem SD og antal forsøgspersoner i dette forsøg.

**Inklusionskriterier**

Deltagelsen i forsøget er forbeholdt patienter med T2D og kontrolpersoner, som ønsker at deltage i eksperimenterne. Ligeledes skal følgende inklusionskriterier opfyldes:

- Type 2 diabetes, dvs. glukosekoncentration af venøs plasma ≥ 11.1 mmol/L 2 timer efter oral glukose tolerance test (OGTT) – gælder kun for T2D-patienter
- Alder: 40-60 år
- Køn underordnet
- Body Mass Index (BMI): 25-40 kg/m^2^
- Skriftligt samtykke før undersøgelsesstart

*Bemærk: i forsøget lægges der ikke op til at overvåge patienternes indtag af mikro- og makronæringsstoffer. Dog vil der, såfremt patienten opfylder inklusionskriterierne, kort blive gennemgået forslag til optimal diabetesdiæt med henblik på at standardisere forsøget så meget som muligt. Herunder anføres de vigtigste ernæringsmæssige anbefalinger for en diabetespatient:*

- *Proteinindtaget bør være 15-20% af det totale energiindtag*
- *Mættet fedtindtag bør være <7% af total mængde kalorier*
- *Erstat mættede fedtsyrer med mono- og polyumættede fedtsyrer*
- *Kolesterolindtag bør være <200 mg/dag*
- *Monitorer kulhydratindtaget som en komponent af glykæmisk kontrol*
- *Undgå høj-protein diæter for at opnå vægttab*
- *Undgå regelmæssig supplement med antioxidanter (f.eks. vitamin C og E, karoten)*
- *Spis ≥ portioner fisk om ugen (ikke kommercielt stegt) for at få n-3 polyumættede fedtsyrer*
- *Minimer indtag af transfedtsyrer*
- *Varier typen af kulhydratindtag (f.eks. frugt, grøntsager, fuldkornsbrød)*
- *Fødevarer med sukrose (traditionel bordsukker) kan erstattes med andre kulhydrater i madplanen*
- *Begræns alkoholforbruget til ≤ 1 genstand/dag for kvinder og ≤ 2 genstande/dag for mænd*

*Kilde: Diabetes Care. 2008;31(Suppl 1):S61-S78*

**Eksklusionskriterier**

- Alder: < 40 år eller > 60 år
- Må ikke være undersøgt med eller have deltaget i andre forsøg, der involver ioniserende stråling indenfor det sidste år.
- Fysisk aktiv, herunder > 1 times moderat intensitetstræning pr. uge
- Insulinbrug
- Akut myokardieinfarkt og ustabil angina, indtil der har været stabil tilstand i mindst ét døgn
- Endocarditis, myocarditis, pericarditis
- Ukontrollable arytmier
- Svær hypertension, herunder systolisk blodtryk>200 mmHg eller diastolisk blodtryk>110 mmHg.
- Graviditet og/eller amning
- Fysisk aktiv, herunder regelmæssig udført motion
- Ansættelse på Institut for Biomedicin eller Institut for Idræt (begge ved AU)
- Neurologisk og/eller psykiatrisk lidelse
- Ekstrem overvægt (BMI>40, idet en højere værdi praktisk vil besværliggøre skanningerne)

*Bemærk: forud for de kliniske forsøg er det vigtigt, at patienten holder pause med kaffe, the, cola og alkohol mindst 24 timer før undersøgelserne, da det kan påvirke målingen af blodgennemstrømningen. Følsomheden og enkelte typer af medicin kan påvirke forløbet af undersøgelsen, hvorfor patienterne vil blive anmodet om at pausere langtidsvirkende nitrater, koffeinholdige præparater/methylxanthiner og dipyridamol henholdsvis 12, 24 og 36 timer før undersøgelsen.*

**Forløb og metodeoversigt**

Dette projekt udføres i samarbejde med Afdelingen for Klinisk Medicin (Klinisk Forskningsernæring, Tage Hansens Gade), Sektion for Idræt på Aarhus Universitet samt Endokrinologisk Afdeling, Aarhus Universitetshospital. På Sektion for Idræt foretages træningsforsøgene (dvs. måling af VO_2peak_ med tilhørende målinger af hjertet med elektrokardiogram, blodtryk og puls, tidskørsler samt flowmedieret blodgennemstrømning). På Endokrinologisk Afdeling foretages DXA-skanninger. På Tage Hansens Gade foretages oral glukose tolerance test (OGTT). På Sektion for Idræt foretages blodgennemstrømningsmåling i hvile omkring forlårsmuskulaturen. I de tilfælde, hvor en stor patientgruppe rekrutteres fra Region Nordjylland udføres OGTT, DXA, blodgennemstrømningsmåling og VO_2peak_ på Hjørring Sygehus, som har lignende faciliteter som i Århus.

Selve HIIT-forløbet vil foregå i et lokalt fitnesscenter. Nedenfor er det forsøgt grafisk at lave to oversigter over det totale undersøgelsesforløb (figur 2 og 3), efterfulgt af en præsentation af de enkelte metoder.

Screeningsbesøget omfatter kontrol af in- og eksklusionskriterier, objektiv undersøgelse inklusiv måling af højde, vægt, blodtryk og puls. Patienter med T2D er på forhånd blevet diagnosticeret med T2D fra Afdeling for Klinisk Medicin med en glukosekoncentration af venøs plasma ≥ 11.1 mmol/L 2 timer efter oral glukose tolerance test (OGTT).

*Grafisk oversigt over forløbet forud for HIIT-forløbet*

**
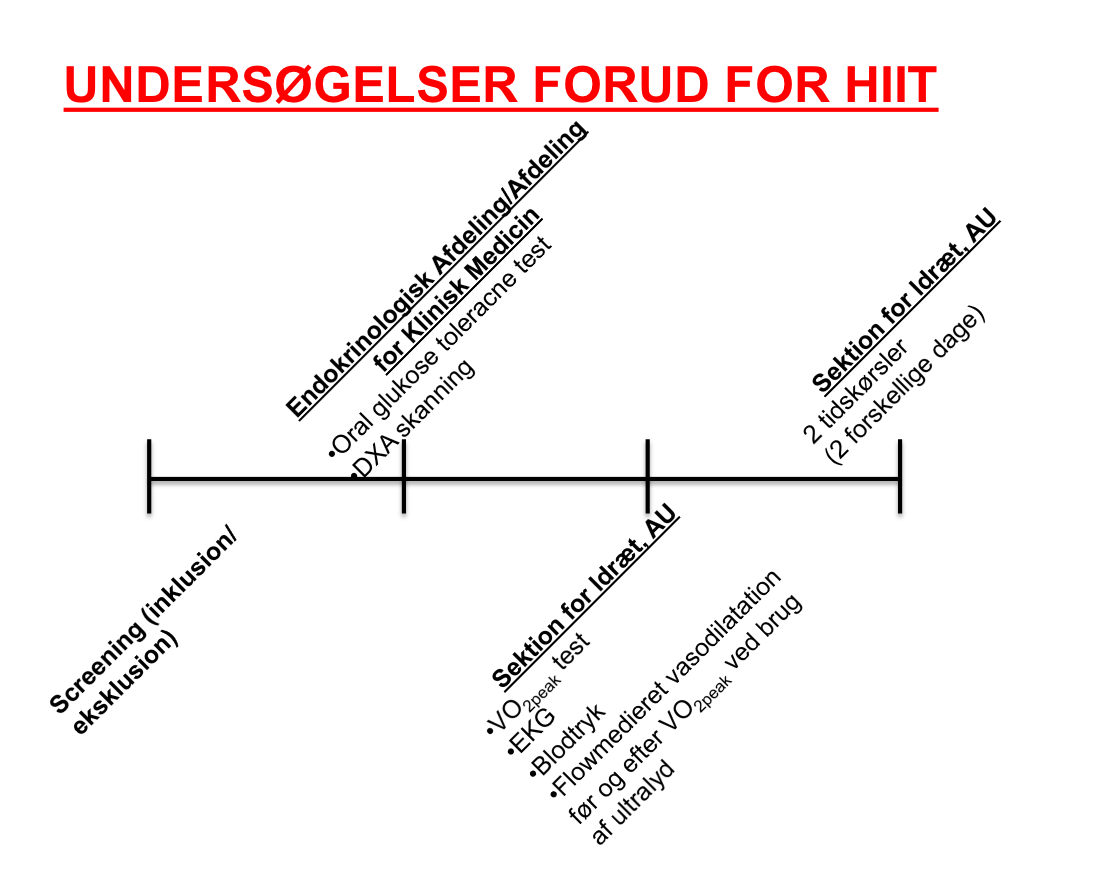
**

**Figur 2:** viser oversigten over de tests, som foretages forud for det 8 uger lange HIIT-forløb. Undersøgelserne forventes at vare ca. 14 dage. Screeningen omfatter kontrol af in- og eksklusionskriterier, dvs. her rekrutteres både T2D-patienter og matchede kontroller.

*Grafisk oversigt over forløbet efter HIIT-forløbet*


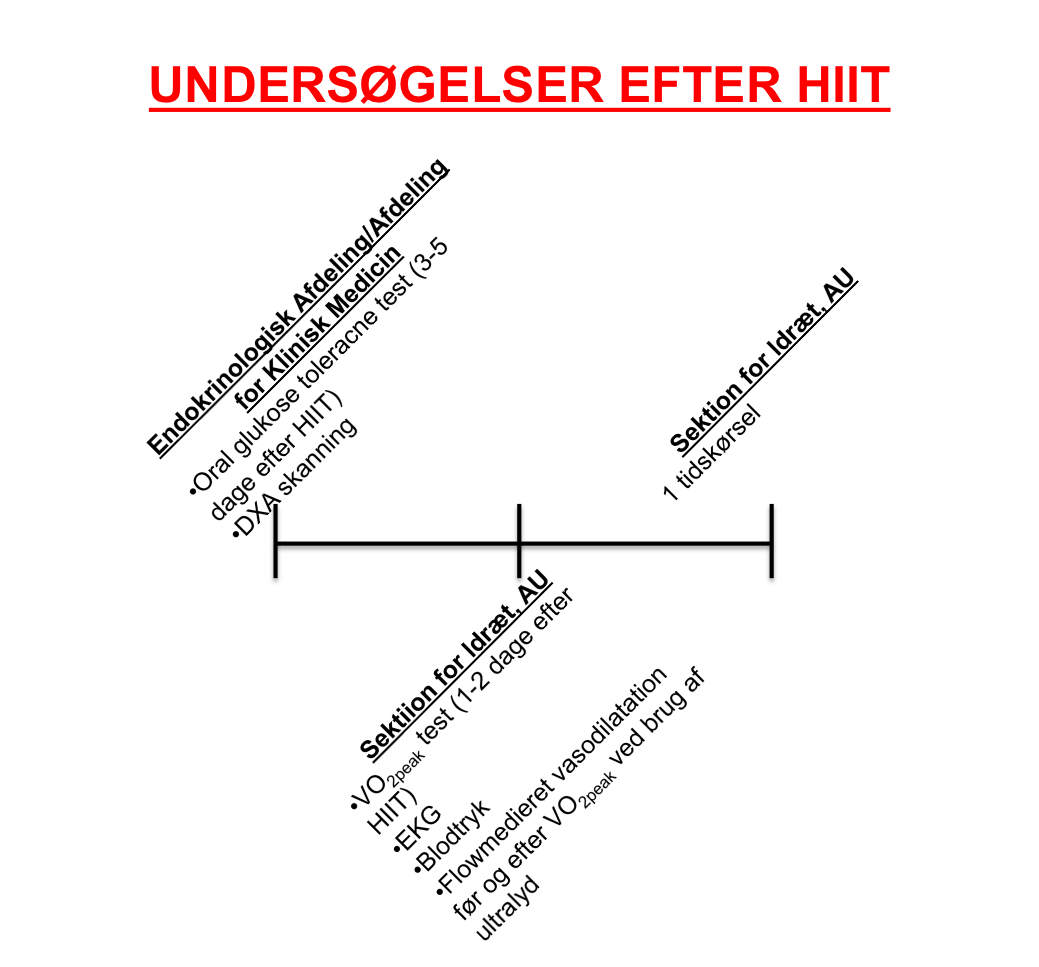


**Figur 3:** viser oversigten over de tests, som foretages efter det 8 uger lange HIIT-forløb. Undersøgelserne forventes at vare ca. 14 dage.

SEKTION FOR IDRÆT, AARHUS UNIVERSITET

*VO_2peak_ test*

VO_2peak_ (dvs. højeste iltoptagelse) optages som den højeste VO_2_ målt i 30 sekunder under testen ved brug af et online respiratorisk gasanalysesystem, såkaldt *Amis 2001* (se figur 4 nedenfor).

Inden forsøgspersonen ankommer tændes testudstyret (Amis 2001) mindst 30 min inden brug. Derefter registres omgivelsernes temperatur, luftfugtighed og tryk. Slangen afkobles fra blandingssækken fra ”gas-inlet” og tilsluttes igen efter målingen. Herefter kalibreres gasmåleren, og en flaske med standardgas (4.00% CO_2_, 16.53% O_2_) benyttes. Flowmeteret kalibreres mht. offset og flow inden hver test. 3-vejsventilen er monteret under kalibreringen.

Når forsøgspersonen er ankommet:

1. Forsøgspersonen vejes og måles og data registreres i ”patient data”
2. Blodtryk måles i liggende position med Omron Intelli Sense M6. Her får forsøgspersonen placeret en manchet omkring venstre overarm, hvorefter et fuldautomatisk oppumpningssystem sikrer, at manchetten pustes op til det nødvendige tryk. Det målte blodtryk registreres i et skema.
3. Pulsbælte føres omkring forsøgspersonens bryst
4. Justering af sadelhøjde og styr (samt eventuel montering af pedaler til cykelsko) foretages – indstillingerne registreres i et skema.
5. Forsøgspersonen varmer op på en Monark ergometercykel 828E i 8-10 min ved opvarmningspuls svarende til ~120-130 slag/min. Samme kadence anvendes under opvarmning som under test (evt. selvvalgt), og en metronom indstilles herefter. Der indstilles en passende bremsekraft.
6. Ansigtsmasken monteres og sluttes tæt til 3-vejsventilen.
7. Testen forklares kort for forsøgspersonen. Forsøgspersonen skal fokusere på at holde kadencen så længe som muligt og først stoppe når han/hun er helt udmattet. Forsøgspersonen skal dog også stoppe, hvis der opstår stærke smerter eller andet ubehag. Forsøgslederen holder desuden øje med forsøgspersonens tilstand under forsøget.

Testproceduren omfatter:

1. Testen påbegyndes når forsøgspersonen når den aftalte kadence. Pulsur samt registreringen af den pulmonære gasudveksling startes samtidigt.
2. Testen udføres som en trappetest med tidsintervaller på 1 min med en startbelastning på 50 watt, hvorefter belastningen øges med 15 watt/min (se f.eks. (19,20)– se endvidere figur 4). Stigningen i intensitet foregår ved progressiv øgning af ”bremsekraften” med fastholdt kadence. Der sigtes på en stigningstakt der gør, at forsøgspersonen kan gennemføre mindst 6 intervaller.
3. Testen fortsætter til forsøgspersonen er helt udmattet. Dvs. at forsøgspersonen ikke kan fortsætte længere eller at kadencen falder mere end 5 *revolutions per minute* (rpm) under det aftalte, og ikke kan genetableres trods gentagne opfordringer fra forsøgslederen. Testen afsluttes med at spørge til årsag til stop (muskulær eller respiratorisk udmattelse eller andet). Som kriterium for veludført test bruges puls og R-værdi (respiratorisk udvekslingskvotient). Sidstnævnte bør være >1.15, idet det indikerer hyperventilation og dermed maksimal udmattelse.
4. Forsøgslederen giver fortløbende verbal motivation til forsøgspersonen.

*Bemærk: under hele forsøget er det let adgang til en defibrillator, som er placeret i samme lokale, som forsøget udføres i. Ligeledes har forsøgslederen altid en mobil på sig i tilfælde af, at en nødsituation måtte opstå.*


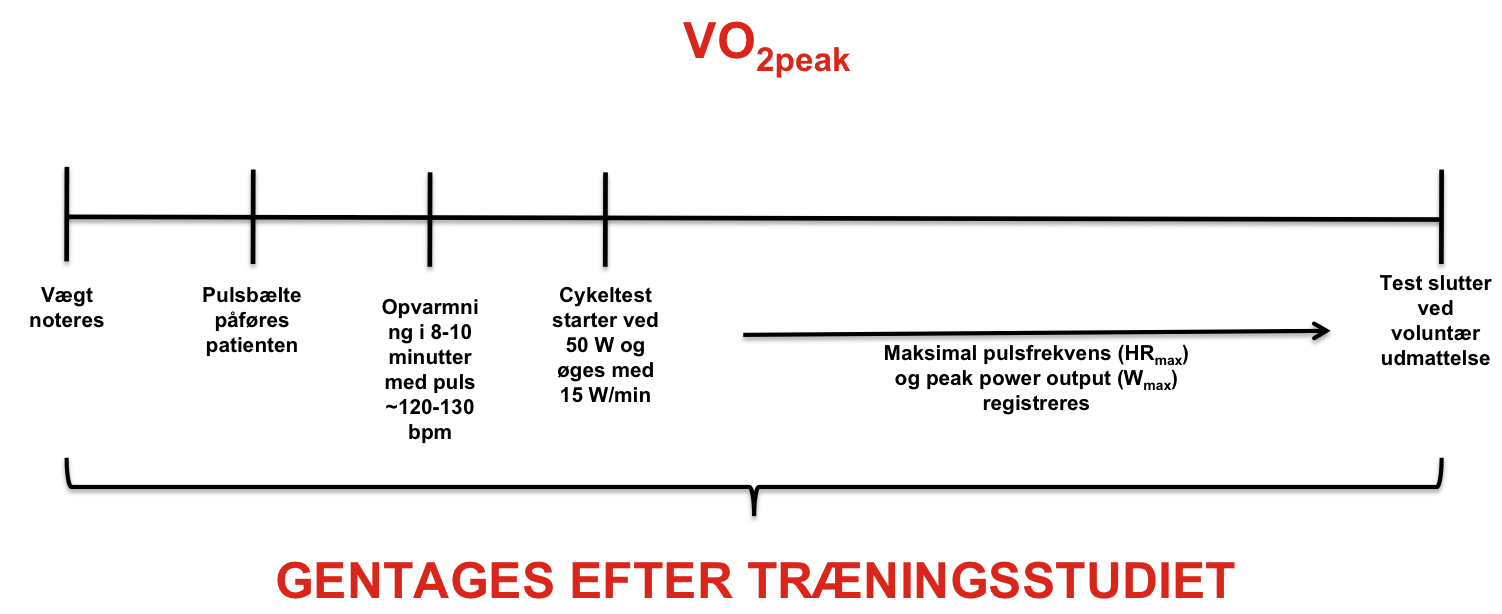


**Figur 4:** viser oversigten over VO_2peak_ testen, som alle forsøgspersoner og patienter udfører før og efter HIIT-studiet. Forsøgene udføres på Institut for Idræt, Aarhus Universitet

Der foretages endvidere 12-ledet elektrokardiogram (EKG)-analyse før og efter VO_2peak_-testen med henblik på at bekræfte fraværet af underliggende kontraindikationer for anstrengende træningsdeltagelse. Således vil der være en læge tilstede, som er i stand til at udføre EKG-målinger.

*Tidskørsler*

To ens tidskørsler (svarende til 5 km) udføres på Monark cykelergometer 828E, hvoraf den hurtigste bruges til senere sammenligning (12). Første tidskørsel anvendes med henblik på at blive familiær med metoden, om end testen stadig skal udføres så hurtig som muligt. Ingen fysiologisk, temporal eller verbal feedback gives, på nær antal kørte km. Anden tidskørsel udføres to dage efter den første; den hurtigste tilbagelagte tid af de to tidskørsler bruges til senere sammenligning (se figur 4). Forsøget udføres af en fysiolog.


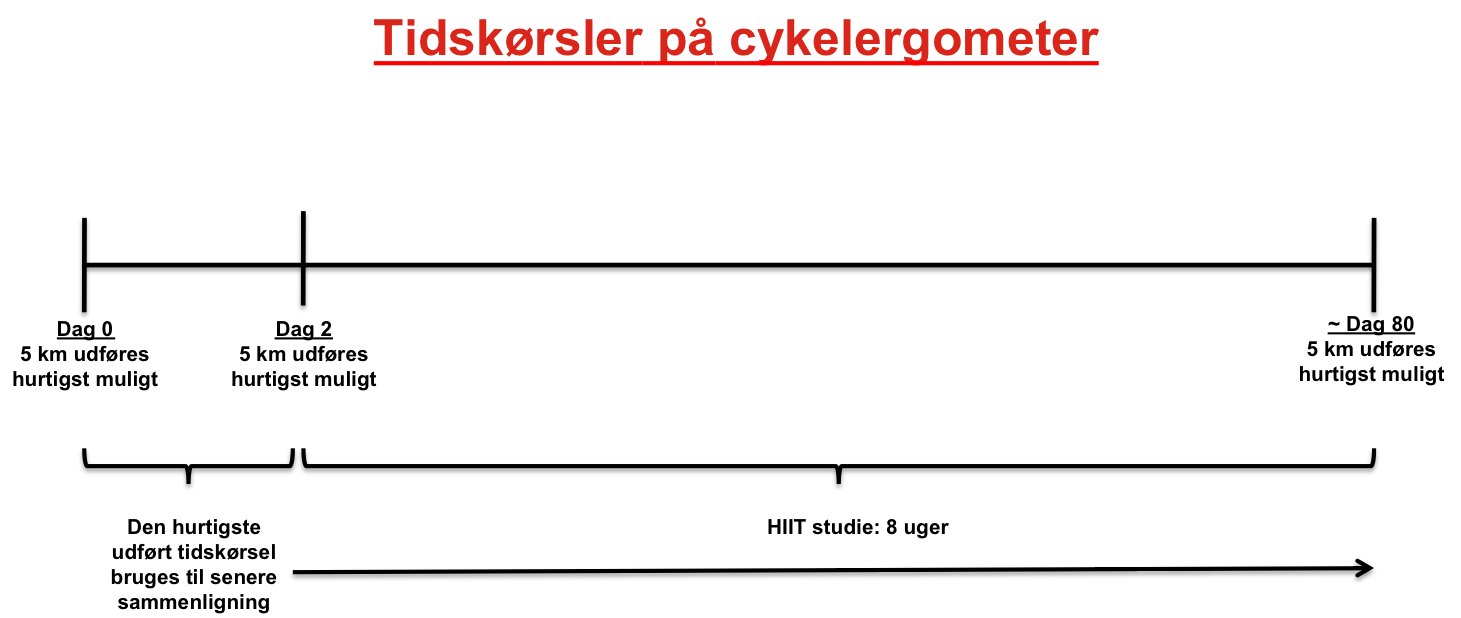


**Figur 5:** viser oversigten over tidskørslerne, hvoraf den hurtigste af de to første bruges til senere sammenligning.

AFDELING FOR KLINISK MEDICIN, TAGE HANSENS GADE

*Oral glukose tolerance test (OGTT)*

En OGTT er en 2.5 timer lang medicinsk test, hvor 75 gram glukose opløses i 100 ml vand (se figur 5). Dette gives til patienten efterfulgt af opsamlinger af venøse blodprøver med henblik på at bestemme, hvor hurtigt glukosen fjernes fra blodet. Patienten møder fastende på Endokrinologisk Afdeling, Aarhus Universitetshospital, hvorefter en venflon anlægges af en læge på underarmen (v. antecubiti) til blodprøvetagningen. Der tages blodprøver 30 og 15 minutter før OGTT samt til tiden 0 (baseline), 10, 20, 30, 45, 60 og 120 min, hvorefter testen afsluttes. Der må ikke spises, drikkes eller ryges under testen. De venøse blodprøver analyseres for glukose, insulin, glukagon, lipider (triglycerider, HDL-, LDL- og totalkolesterol) samt cytokiner (TNF-α, IL-6, IL-10 og c-reaktivt peptid). Centrifugering bruges til at separere plasma fra de røde blodlegemer og vil blive opbevaret ved -20°C frem mod analyserne. OGTT udføres af erfarent personale, herunder læge, bioanalytiker og fysiologer. Glukoseopløsningen bestilles ved Hospitalsapoteket på Aarhus Universitetshospital, og opbevares i et køleskab indtil brug.


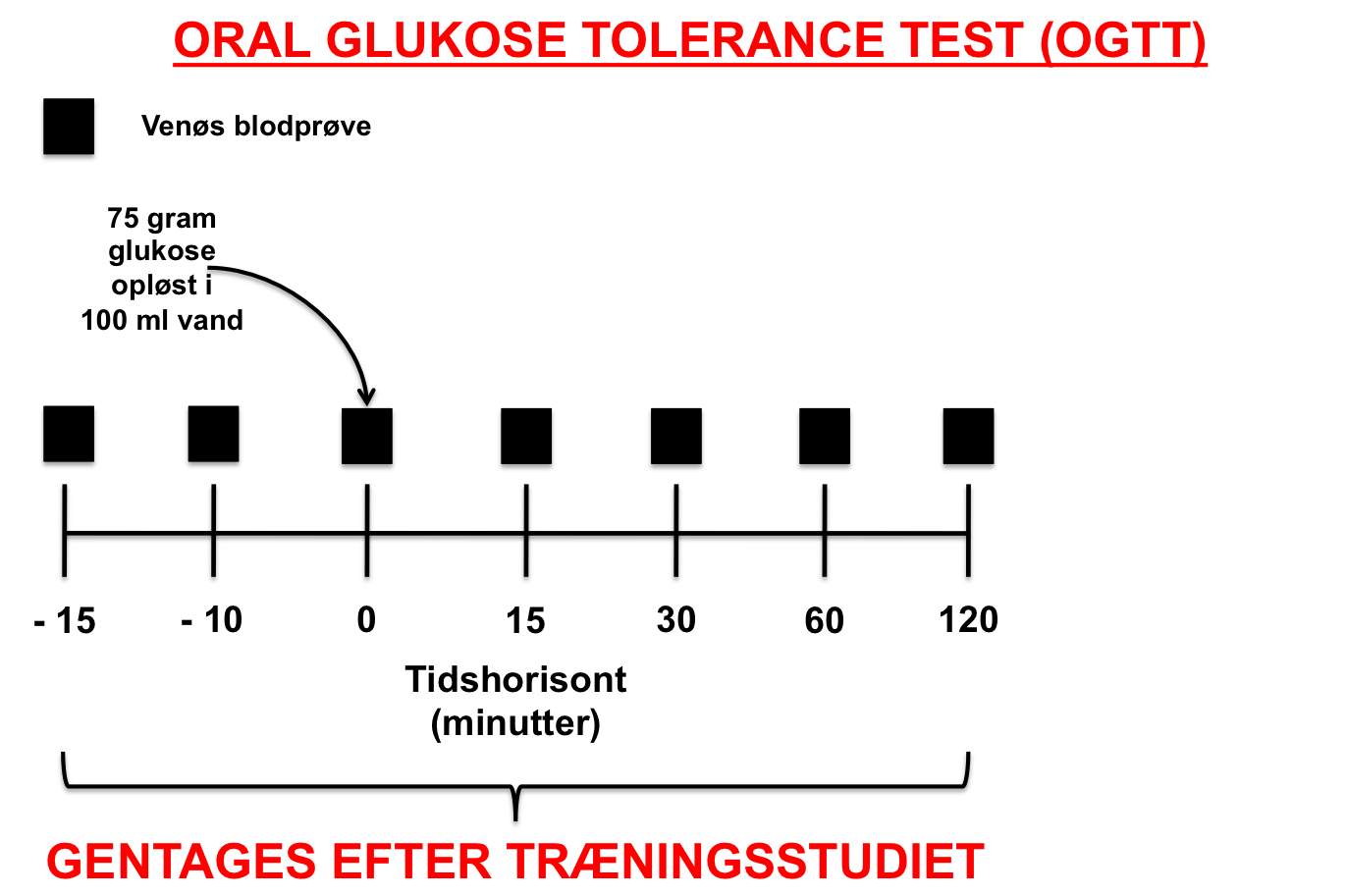


**Figur 6:** viser oversigten over oral glukose tolerance test (OGTT). Den udføres før og efter den 8 uger lange superviseret træningsperiode. Hver sorte markering angiver, hvornår blodprøverne tages.

ENDOKRINOLOGISK AFDELING, AARHUS UNIVERSITETSHOSPITAL

*Dual-emission X-ray absorptiometry (DXA)*

Dette apparat (Prodigy, GE Healthcare) bruges til at måle fedtfri masse, fedtmasse og kropsvægt, og betragtes som et validt og præcist måleinstrument (21). DXA skanning er en præcis metode til at måle kropssammensætningen, og skal bruges til at validere de antropometriske mål fra undersøgelsen. Forsøgspersonen placeres på en briks i liggende position, og en skanner-arm bevæger sig hen over kroppen. Skanningen er uden ubehag og varer ca. 15 min, og foretages henholdsvis før og efter træningsinterventionen. Tøj med metalknapper, spænder, lynlås samt smykker eller lignende bedes undgået (kan evt. tages af under undersøgelsen). DXA-skanningen foretages af erfarent personale. Umiddelbart efter OGTT og DXA-skanningen får forsøgspersonerne et let måltid mad.

SEKTION FOR IDRÆT, AARHUS UNIVERSITET

*Flowmedieret vasodilatation (FMD)*

FMD er en non-invasiv procedure og anvendes med henblik på at måle vasodilatorisk funktion *in vivo*. FMD menes i høj grad at reflektere endothelial-afhængig og overvejende nitrodoxid-medieret arteriefunktion og bruges som surrogat for vaskulært helbred.

Der anvendes Philips iU22 ultralydsapparat og blodtryksmanchet, der kan opretholde et tryk ≥ 200 mmHg i 5 minutter. Metoden er standardprocedure ved MEA.

Når forsøgspersonen er ankommet:

- Hvile i ≥ 20 minutter i mørkt lokale. Forsøgspersonen placeres på en briks i liggende stilling.
- FMD tests (within-subject) udføres på samme tidspunkt før og 60 minutter efter VO_2peak_-tests og samme forløb gentages efter HIIT-forløbet.
- Blodtryksmanchetten placeres distalt i forhold til den arterie, der måles og pumpes op i 5 minutter (såkaldt reaktiv hyperæmi).
- Forsøgspersonerne er fastende.
- Det er vigtigt, at forsøgspersonen holder pause med kaffe, the, cola og chokolade mindst 24 timer før undersøgelsen, da det kan påvirke målingen af blodgennemstrømningen. Ligeledes er det vigtigt, at forsøgspersonen ikke har trænet mindre end 24 timer forud for forsøget. Ydermere skal det understreges, at forsøgspersonen forud for forsøget benytter sig af offentlige transportmidler eller privat kørsel (dvs. cykling eller løb vil ekskludere forsøgspersonen fra FMD-test).
- T2D-patienterne overvåges nøje i forhold til om de følger et fastsat medicinprogram – således kan svingende medicinering (både tid og brug) have stor betydning for udfaldet af FMD.
- Præmenstruelle kvinder bør vurderes fra dag 1-7 i menstruationscyklussen.

Protokollen, som forsøgspersonen skal gennemgå:

- Baseline diameter af a. poplitea undersøges før blodtryksmanchetten pumpes op (baseline). Der måles i 1 minut.
- Blodtryksmanchetten pumpes op i 5 minutter (reaktiv hyperæmi FMD/shear stress) til et tryk ≥ 200 mmHg (suprasystolisk tryk), hvorefter manchettrykket øjeblikkeligt nedbringes til 0 mmHg. I denne fase måles blodgennemstrømningen (ml/min) af a. poplitea indenfor 15 sek. og diameteren af a. poplitea ved 45, 90, 180 og 300 sek.
- Målingen af aktiv hyperæmi foretages i ≥ 5 minutter for xx (jf . figur 7)


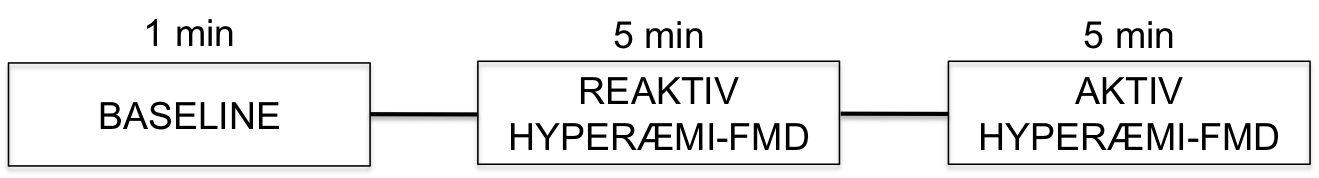
Målinger, der er nødvendige for FMD:

**Figur 8:** oversigt over FMD-test. Indledningsvist baseline, hvor arteriens blodgennemstrømning og diameter måles efterfulgt af en 5 minutters periode (reaktiv hyperæmi), hvor arterien strammes af en blodtryksmanchet (suprasystolisk tryk skabes) og slutteligt af en 5 minutters periode (aktiv hyperæmi), hvor manchetten løsnes. Det er forskellen imellem reaktive og aktive hyperæmi-FMD faser, der måles på, og som kvantitativt kan fortælle om forsøgspersonens endotheliale (dys)funktion før og efter HIIT-forløb. I tilfælde af forbedret endothelial funktion afspejles dette i en øget FMD.

- Shear rate (reaktiv hyperæmi FMD) = gennemsnitlig blodgennemstrømning/kardiameter
- FMD = a. poplitea_peak diameter_ – a. poplitea_baseline diameter_/ a. poplitea_baseline diameter_

*Såfremt de allerede rekrutterede forsøgspersoner accepterer de nye ændringer (nu non-invasiv procedure udført i Århus C., tidligere invasiv procedure udført i København) bliver de indkaldt til at få målt deres individuelle blodgennemstrømning, hvilket vil tage 2 timer/forsøgsdeltager.*

LOKAL FITNESSCENTER

*Træningsprogram*

Forsøgspersonerne udfører superviseret højintens intervaltræning 3 gange om ugen over 8 uger på cykelergometer (Keiser, M3) og lokale stilles til rådighed hos SeierFitness, Aarhus C. Forud for den 8 uger lange træningsperiode møder forsøgspersonen ind til en gennemgang af cyklens opsætning, hvor han/hun får mulighed for at vænne sig til cyklen (herunder belastning, højdeindstilling og styrindstilling). Under træningsperioden udfører forsøgspersonen et individuelt fastlagt program, som hver gang omfatter 10×60 sek. arbejde (afbrudt af 60 sek. pause med en belastning svarende til ~50 watt) svarende til en kraft, der under VO_2peak_-testen fremkalder ~90% af den maksimale hjertefrekvens (se figur 3 nedenfor). Tidsforbruget per session forventes at vare ~25 minutter. Under alle træningspas bærer forsøgspersonerne TEAM2 WearLink+ (Polar, Kempele, Finland) med henblik på at måle deres hjertefrekvens, der efterfølgende overføres til pc. Alle træningstimer køres i fællesskab med en spinningsinstruktør samt vil der altid være en træningsvejleder tilstede, som kontrollerer forsøgspersonernes kørsel, og at denne stemmer overens med det planlagte HIIT program. Under testen gives løbende feedback med henblik på at få forsøgspersonen til at cykle så hurtigt som muligt.


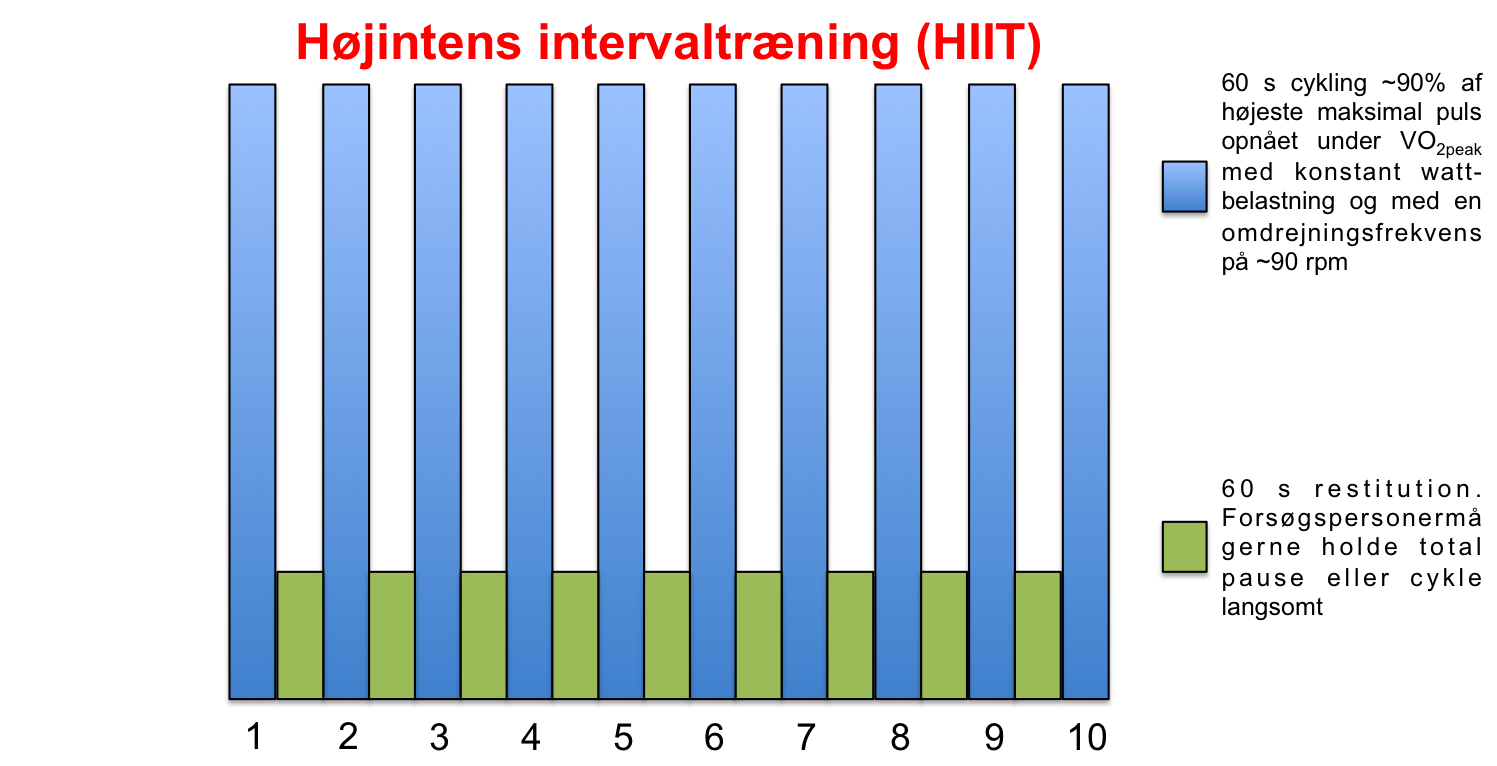


**Figur 8:** viser oversigten over en enkelt HIIT forsøgsgang, hvilket skal udføres over en 8 uger lang træningsperiode.

INDIVIDUELLE MÅLINGER

*Målinger af kapillær blodglukose koncentrationen:*

Patienten vil få udleveret et apparat til at kvantificere kapillær blodglukosemåling, som bestemmes hver morgen umiddelbart efter opvågning (postprandiel fase) og før indtag af morgenmad. Til apparatet udleveres også strimler og fingerprikker. Data herfra registreres i et udleveret ark, hvori patienten indtaster den målte blodglukose koncentration, og sendes til den forsøgsansvarlige. Således skal patienten foretage 56 hjemmemålinger. Forud for dette instrueres patienten nøje i brugen af systemet og patienten foretager en enkelt priktest selv for at kontrollere, at patienten selv er i stand til at kunne udføre målingen.

## Effektparametre

- Måling af hvilende vasodilatation før og efter HIIT ved brug af FMD
- Metabolisme-differenser (glukose og insulin)
- HIIT som træningsstrategi
- En række inflammatoriske markører (interleukiner, cytokiner, m.fl.)

## Statistiske overvejelser

Under den statistiske gennemgang af data præsenteres disse som gennemsnit ± S.E.M. Data fra blodprøverne fra oral glukose tolerance test (foretaget før og efter HIIT) analyseres ved brug *two-way repeated measures ANOVA* med *post hoc* Student Newman-Keuls tests. Forskelle mellem før HIIT-data og efter HIIT-data af henholdsvis VO_2peak_, tidskørsler samt blodgennemstrømningsmålinger under et-bens knæ-ekstensor arbejde og VO_2peak_ (målt med thermodilution og CEUS) analyseres med *Student’s paired t-test*. *Pearson’s correlation coefficient* vil blive brugt til at vurdere bivariate korrelationer mellem baseline værdier, og ændringer af følgende variabler: metaboliske markører, blodgennemstrømning (flowmedieret vasodilatation), de forskellige træningsfysiologiske tests samt målinger fra skanninger. P-værdier under 0.05 vil blive antaget for statistisk signifikante. Statistikprogrammerne STATA (til beregning) og Prism GraphPad (til visuel fremstilling) anvendes.

## Risici og bivirkninger

Der er minimale gener forbundet med blodprøveudtagning, og da der gennem hele forsøgsperioden udtages omtrent 100 ml venøst blod (~1/4 af en almindelig bloddonation), udgør dette derfor ingen risiko for forsøgspersonen. Venflonanlæggelse foregår under sterile forhold. Anlæggelse af katetre er standardprocedurer ved Afdeling for Klinisk Medicin (Tage Hansens Gade). Forsøgspersonerne rådes til at undgå intenst fysisk aktivitet om aftenen på selve forsøgsdagen. Et mindre hæmatom som følge af venflonanlæggelsen kan forekomme og give ømhed og gener i dagene umiddelbart efter forsøget.

Der er i forbindelse med udførsel af DXA-skanninger ingen fare for deterministiske skader, men en risiko for stokastiske skader. Således udgør den stokastiske strålebelastning under DXA-skanningerne 0.02-0.05 mSv pr. undersøgelse (~0.04-0.10 mSv). Til sammenligning er den årlige baggrundsstråling i Danmark 3.0 mSv, og mindre end dosisgrænsen (20 mSv pr. år for arbejdstagere over 18 år). I Danmark er risikoen for at få uhelbredelig kræft 25%. Med skanningerne i dette forsøg svarer det til, at der er 25.0005% øget risiko for kræft (dvs. 0.0005%). Det vurderes derfor, at eventuelle risici og bivirkninger opvejes af de forventelige fordele ved deltagelse i forsøget.

Ved alvorlige hændelser, som er en følge af projektet, vil den forsøgsansvarlige indberette de oplysninger, Videnskabsetisk Komité (Region Midtjylland) måtte anmode om. Hvis der optræder alvorlige hændelser eller bivirkninger indsendes en liste til Videnskabsetisk Komité (Region Midtjylland).

## Videnskabsetisk redegørelse

Selve ultralydsundersøgelse er ikke forbundet med risici. Risikoen, som tilføres patienten ved intraarteriel administration af ultralydskontraststof vurderes som værende meget lav. Det samme gælder DXA-skanningerne. Undersøgelsen udføres i overensstemmelse med Helsinki II deklarationen. Den Videnskabsetiske Komité for Region Midtjylland ansøges om projekttilladelse. Deltagelse i undersøgelsen er frivillig. Enhver deltager kan til enhver tid udgå af studiet, hvis vedkommende ønsker dette, og deltageren kan ikke afkræves nogen forklaring desangående.

Projektet involverer både T2D patienter og raske, myndige, habile forsøgspersoner, der deltager frivilligt efter fyldestgørende mundtlig og skriftlig information og skriftligt samtykke. Der indgår således heller ikke patientdata eller lignende. Deltagelse er frivillig, og man kan til hver en tid afbryde forsøget og forlade undersøgelsen uden at skulle begrunde det.

Deltagerne vil være fuldstændigt anonyme, og personlige oplysninger vil blive behandlet fortroligt. Forsøgsdeltagerne vil til enhver tid have mulighed for at få yderligere information om projektet ved henvendelse til den forsøgsansvarlige.

## Anmeldelse til Datatilsynet

Studiet gennemføres på Endokrinologisk Afdeling på Aarhus Universitetshospital, Center for Inflammation og Metabolisme samt på Institut for Idræt. Ingen af de medvirkende i forsøget har økonomiske interesser knyttet til private erhvervsvirksomheder eller fonde, der indgår i forsøget. Projektet overholder lov om behandling af personoplysninger, og projektet anmeldes til datatilsynet. Der er d. 28/01/2013 foretaget en anmeldelse til regionens fællesanmeldelse til Datatilsynet. Forsøget vil blive udført i fuld overensstemmelse med loven om behandling af personoplysninger.

## Økonomisk støtte

MEA står for udgifterne i forbindelse med indkøb af medicinsk udstyr. De forsøgsansvarlige har ingen økonomisk interesse i forsøget. Der vil blive søgt støtte fra Diabetesforeningen, Hjerteforeningen og Århus Universitets frie forskningsmidler. Hertil kommer, at der også forsøges at blive søgt hos andre relevante fonde.

## Økonomisk vederlag til forsøgsdeltagere

Der ydes et honorar på i alt 3000 kr. til forsøgspersonerne, som opgives til SKAT som B-indkomst. Der ydes godtgørelse for kørsels- og transportudgifter efter statens gældende takster i forbindelse med undersøgelserne (2.10 kr/km). MEA står for omkostningerne ved abonnementerne til et træningscenter.

**Forsikring**

Patienterne dækkes i henhold til patientforsikringsloven.

**Offentliggørelse**

Dette forsøg danner grundlaget for Søren Møller Madsens ph.d.-projekt, hvormed det forsøges at validere CEUS. I den forbindelse vil publikation af resultater fra projektet koordineres af den projektansvarlige i overensstemmelse med retningslinjer angivet i ’Vejledninger for god Videnskabelig Praksis’, Udvalget Vedrørende Videnskabelig Uredelighed, 1998.

Både konklusive og inkonklusive resultater af undersøgelserne vil løbende blive offentliggjort i internationale videnskabelige tidsskrifter, f.eks. Journal of Physiology.

Positive såvel som negative resultater af undersøgelserne vil løbende blive offentliggjort i internationale videnskabelige tidsskrifter, f.eks. Journal of Physiology.

**Tidsplan**

Protokol færdig: Januar 2013

Godkendelse fra Videnskabsetisk Komité: Februar 2013

Forsøgsstart April/maj 2013

Forsøgsafslutning Juni 2014

Dataanalyse og artikelskrivning Løbende

Publikation/offentliggørelse Maj 2015

## Forløb for rekruttering og indhentning af samtykke

Første kontakt med rekruttering af forsøgsdeltagere: Forsøgsdeltagerne hverves efter opslag på www.forsøgsperson.dk, sundhedscentre i Region Nordjylland og Regions Sønderjylland, på Aarhus Universitetshospital og i aviserne Århus Onsdag og Nordjyske. Rekrutteringen foregår ved, at der laves opslag om forsøgets indhold, herunder projektets mål, hvorledes undersøgelsen udføres og hvilke risici, der er forbundet med at deltage i forsøget (se Skriftlig deltagerinformation).

I god tid inden undersøgelsen, typisk mellem to dage og en uge før, orienteres den frivillige forsøgsperson af projektansvarlige mundtligt om undersøgelsens formål, indhold og risici, og om at vedkommende på et hvert tidspunkt kan trække sig ud af undersøgelsen. Ved samme lejlighed udleveres det skriftlige materiale (se Skriftlig deltagerinformation). På dagen for selve undersøgelsen gentages den mundtlige orientering, og efter besvarelse af eventuelle spørgsmål indhentes forsøgspersonens skriftlige samtykke (se Samtykkeerklæring). Informationsmøderne vil finde sted på Medicinsk Endokrinologisk Afdeling (MEA) på projektansvarliges kontor eller i et undervisnings- eller mødelokale. Ved større grupper arrangeres mødes på det lokale sundhedscenter.

Det gøres klart i det skriftlige materiale til hvervning at forsøgsdeltagerne har ret til at tage en bisidder med til informationsmødet.

Skriftligt samtykke indhentes umiddelbart før selve undersøgelsen. Forsøgsdeltageren har således haft mellem to dage og en uges betænkningstid.

# Referencer

(1) Shaw JE, Sicree RA, Zimmet PZ. Global estimates of the prevalence of diabetes for 2010 and 2030. Diabetes Res Clin Pract 2010 Jan;87(1):4-14.

(2) Dor Y, Brown J, Martinez OI, Melton DA. Adult pancreatic beta-cells are formed by self-duplication rather than stem-cell differentiation. Nature 2004 May 6;429(6987):41-46.

(3) Georgia S, Bhushan A. Beta cell replication is the primary mechanism for maintaining postnatal beta cell mass. J Clin Invest 2004 Oct;114(7):963-968.

(4) Jessen N, Goodyear LJ. Contraction signaling to glucose transport in skeletal muscle. J Appl Physiol 2005 Jul;99(1):330-337.

(5) Porte D,Jr, Kahn SE. Beta-Cell Dysfunction and Failure in Type 2 Diabetes: Potential Mechanisms. Diabetes 2001 Feb;50 Suppl 1:S160-3.

(6) Rhodes CJ. Type 2 diabetes-a matter of beta-cell life and death? Science 2005 Jan 21;307(5708):380-384.

(7) Womack L, Peters D, Barrett EJ, Kaul S, Price W, Lindner JR. Abnormal skeletal muscle capillary recruitment during exercise in patients with type 2 diabetes mellitus and microvascular complications. J Am Coll Cardiol 2009 Jun 9;53(23):2175-2183.

(8) Thijssen DH, Black MA, Pyke KE, Padilla J, Atkinson G, Harris RA, et al. Assessment of flow-mediated dilation in humans: a methodological and physiological guideline. Am J Physiol Heart Circ Physiol 2011 Jan;300(1):H2-12.

(9) Andersen P, Saltin B. Maximal perfusion of skeletal muscle in man. J Physiol 1985 Sep;366:233-249.

(10) Nyberg M, Jensen LG, Thaning P, Hellsten Y, Mortensen SP. Role of nitric oxide and prostanoids in the regulation of leg blood flow and blood pressure in humans with essential hypertension: effect of high-intensity aerobic training. J Physiol 2012 Mar 15;590(Pt 6):1481-1494.

(11) Currie KD, McKelvie RS, Macdonald MJ. Flow-mediated dilation is acutely improved after high-intensity interval exercise. Med Sci Sports Exerc 2012 Nov;44(11):2057-2064.

(12) Gibala MJ, Little JP, van Essen M, Wilkin GP, Burgomaster KA, Safdar A, et al. Short-term sprint interval versus traditional endurance training: similar initial adaptations in human skeletal muscle and exercise performance. J Physiol 2006 Sep 15;575(Pt 3):901-911.

(13) Gibala MJ, McGee SL. Metabolic adaptations to short-term high-intensity interval training: a little pain for a lot of gain? Exerc Sport Sci Rev 2008 Apr;36(2):58-63.

(14) Burgomaster KA, Hughes SC, Heigenhauser GJ, Bradwell SN, Gibala MJ. Six sessions of sprint interval training increases muscle oxidative potential and cycle endurance capacity in humans. J Appl Physiol 2005 Jun;98(6):1985-1990.

(15) Burgomaster KA, Heigenhauser GJ, Gibala MJ. Effect of short-term sprint interval training on human skeletal muscle carbohydrate metabolism during exercise and time-trial performance. J Appl Physiol 2006 Jun;100(6):2041-2047.

(16) Harmer AR, McKenna MJ, Sutton JR, Snow RJ, Ruell PA, Booth J, et al. Skeletal muscle metabolic and ionic adaptations during intense exercise following sprint training in humans. J Appl Physiol 2000 Nov;89(5):1793-1803.

(17) Saltin B, Nazar K, Costill DL, Stein E, Jansson E, Essen B, et al. The nature of the training response; peripheral and central adaptations of one-legged exercise. Acta Physiol Scand 1976 Mar;96(3):289-305.

(18) Babraj JA, Vollaard NB, Keast C, Guppy FM, Cottrell G, Timmons JA. Extremely short duration high intensity interval training substantially improves insulin action in young healthy males. BMC Endocr Disord 2009 Jan 28;9:3.

(19) Little JP, Gillen JB, Percival ME, Safdar A, Tarnopolsky MA, Punthakee Z, et al. Low-volume high-intensity interval training reduces hyperglycemia and increases muscle mitochondrial capacity in patients with type 2 diabetes. J Appl Physiol 2011 Dec;111(6):1554-1560.

(20) Hood MS, Little JP, Tarnopolsky MA, Myslik F, Gibala MJ. Low-volume interval training improves muscle oxidative capacity in sedentary adults. Med Sci Sports Exerc 2011 Oct;43(10):1849-1856.

(21) Owens S, Gutin B, Allison J, Riggs S, Ferguson M, Litaker M, et al. Effect of physical training on total and visceral fat in obese children. Med Sci Sports Exerc 1999 Jan;31(1):143-148.

**Protokolresume**

**Projekttitel:** *Undersøgelse af blodgennemstrømningen og glukosemetabolismen i forlårsmuskulaturen hos patienter med type 2 diabetes og matchede kontrolpersoner – effekten af 8 ugers kontrolleret høj intens intervaltræning*

**Investigator:** *Søren Møller Madsen, cand.scient. (fysiologi) og ph.d.-studerende*

**Forsøgsansvarlige:** *Per Bendix Jeppesen, cand.scient., ph.d.*

**Forsøgssteder:** *1) Sektion for Idræt, Dalgas Avenue 4, 8000 Aarhus C. 2) Medicinsk Afd. M, Århus Sygehus, Nørrebrogade 44, 8000 Aarhus C. 3) Afdeling for Klinisk Medicin (Klinisk Ernæringsforskning), Tage Hansens Gade 2, 8000 Århus C.*

**Formål og baggrund**

Det overordnede formål med nærværende studie er at undersøge blodgennemstrømningen i forlårsmuskulaturen og sukkerstofskiftet før og efter 8 uger med høj intens intervaltræning (HIIT) på cykelergometer hos patienter med type 2 diabetes (T2D) og matchede kontrolpersoner. Følgende parametre indgår i vurderingen heraf:

- Sukkerstofskiftet
- Muskel- og fedtmassen
- Blodgennemstrømningen i hvile omkring forlårsmuskulaturen
- Iltoptagelsestest på cykelergometer samt 5 km tidskørsel

Type 2 diabetes er blandt verdens hurtigst voksende livsstilsygdomme. Alene i Danmark er der ifølge Sundhedsstyrelsen ca. 245.000 diagnosticerede type 2 diabetes patienter. Hertil kommer, at det vurderes, at lige så mange menes at have type 2 diabetes uden at have kendskab til det. Derudover har op til 750.000 danskere begyndende diabetiske komplikationer. Type 2 diabetes udgør også en sundhedsmæssig risiko i resten af verden; i 2010 mentes 285 millioner at have type 2 diabetes, og dette forventes at øge eksplosivt med op til 439 millioner i 2030. Dermed fremstår type 2 diabetes som en af det 21. århundredes største globale sundhedsudfordringer.

Type 2 diabetes giver anledning til lavere sukkeroptag i skeletmuskulaturen sammenlignet med ikke-diabetiske individer. Genetik, fedme og fysisk inaktivitet er tre vigtige faktorer, der ligger til grund herfor. Samtidig med udviklingen af type 2 diabetes, kan der opstå en række andre hjerte-kar-sygdomme, herunder f.eks. forøget blodtryk, blodprop i hjertet, småkarssygdomme i organer og ben. Foruden ovenstående type 2 diabetes karakteristika, menes sygdommen også at give anledning til ændret – og i nogle tilfælde nedsat – blodgennemstrømning i musklerne. Netop evnen til blodgennemstrømningen i både store og små kar hos type 2 diabetes patienter ønsker vi at undersøge nærmere.

Der har længe ikke været enighed om, hvilken specifik træningsstrategi, der er nødvendig for at tilvejebringe ovenstående helbredsfordele for type 2 diabetes patienter; i særdeleshed med hensyn til forholdet mellem mængde og intensitet. Høj intens intervaltræning er en træningsstrategi, som omfatter korte anstrengende omgange, hver individuelt efterfulgt af en pause, der er tilstrækkelig lang til, at forsøgspersonen er i stand til at komme sig med henblik på at kunne udføre tilsvarende maksimale anstrengelse efterfølgende. I den forbindelse er det blevet forsøgt at udvikle en træningsprotokol til fordel for type 2 diabetes patienter og stillesiddende individer. Træningsprotokollen omfatter 10×60 sekunders intervaller afbrudt af 60 sekunders pause mellem hvert interval, og med en arbejdsintensitet svarende til en kraft, der under en iltoptagelsestest fremkalder ca. 90% af den maksimale hjertefrekvens.

Projektet involverer både type 2 diabetes patienter og raske, myndige, habile forsøgspersoner i alderen 40-60 år. I alt ansøges der om at inkludere 34 forsøgspersoner (17 type 2 diabetes patienter og 17 kontrolpersoner). Patienter med type 2 diabetes er i forvejen blevet diagnosticeret af egen læge. Desuden gælder det, at samtlige forsøgspersoner har et body mass index mellem 25-40 kg/m^2^. Køn er underordnet. Mulige forsøgsdeltagere kan ekskluderes, såfremt de har ustabil hjertesygdom eller alvorlig lungesygdom. Ydermere ekskluderes man, hvis man er gravid eller ammer eller er kraftig overvægtig (body mass index>40 kg/m^2^). Desuden må forsøgspersonen ikke være undersøgt med eller have deltaget i andre forsøg, der involver ioniserende stråling (f.eks. CT-skanning) indenfor det sidste år. Hertil kommer, at insulinbrug og moderat intensitetstræning i mere end 1 time pr. uge ekskluderer forsøgspersonen.

Metodisk benyttes flere forskellige apparaturer til at belyse forsøgspersonernes helbredsmæssige status:

- Maksimal iltoptagelsestest samt tidskørsler (5 km hurtigst muligt) på cykelergometer. Foregår på Sektion for Idræt, Aarhus Universitet
- Sukkerbelastningstest og måling af kropssammensætning. Under sukkerbelastningstesten vil der blive taget blodprøver. Foregår på Endokrinologisk Afdeling (Medicinsk Afdeling M), Aarhus Universitetshospital
- Blodgennemstrømningsmåling i hvile ved brug af ultralyds doppler skanner før og efter maksimal iltoptagelsestest og foregår på Sektion for Idræt, Aarhus Universitet.

Ovenstående undersøgelser vil finde sted umiddelbart før og efter det 8 ugers lange høj intens intervaltræningsforløb. Undersøgelserne strækker sig over i alt 7 dage (4 dage før og 3 dage efter træningsforløbet). Hertil kommer, at forsøgspersonerne i en 8 uger lang periode møder ind 3 gange ugentligt ved et lokalt fitnesscenter. Hver gang bliver forsøgspersonen instrueret af en spinningsinstruktør til at køre et planlagt program, dvs. høj intens intervaltræning med 10×60 sek. med en belastning, der svarer til ca. 90% af deres individuelle maksimale pulsfrekvens. Efter hvert interval er der 60 sekunders hvile.

Forsøget er ikke forbundet med nogen kendte risici eller gener. Der er meget minimal strålingsfare ved måling af kropssammensætningen – risikoen for at få kræft ved deltagelse i forsøget øges med 0.0005%. Ultralydsundersøgelserne er uden bivirkninger, risici og ulemper. Der er minimale gener forbundet med blodprøveudtagning under sukkerbelastningstesten. En mindre blodansamling som følge af katetret kan forekomme og give ømhed og gener i dagene umiddelbart efter forsøget. Forsøgspersonerne rådes til at undgå intenst fysisk aktivitet om aftenen på selve forsøgsdagen.

Der ydes dog et honorar på i alt 3000 kr. til forsøgspersonerne, som opgives til SKAT som B-indkomst. Ydermere godtgøres der for transporten til og fra Århus på de dage, hvor laboratorieforsøgene udføres. Desuden dækker Aarhus Universitetshospital for omkostningerne til et lokalt fitnesscenter.

Forsøgsdeltagerne hverves efter opslag på www.forsøgsperson.dk, sundhedscentre i Region Nordjylland og Regions Sønderjylland, på Aarhus Universitetshospital og i aviserne Århus Onsdag og Nordjyske.

En deltagelse i dette forsøg har en vigtig forskningsmæssig nytte, idet det er med til at forstå reguleringen af blodgennemstrømningen i store og mindre kar hos type 2 diabetes patienter og kontrolpersoner, både i hvile og under fysisk aktivitet. Hvis vore antagelser om forstyrret blodgennemstrømning hos type 2 diabetes patienter viser sig at være rigtige, vil vi på sigt kunne bruge vore erfaringer til at udvikle et diagnostisk værktøj, der kan benyttes til udredning af mange hjerte/kar relaterede sygdomme. Der er dog ingen behandlingsmæssig gevinst ved at deltage i forsøget.

# Skriftlig deltagerinformation

## Deltagerinformation om deltagelse i et videnskabeligt forsøg

**Forsøgets titel:** *Undersøgelse af blodgennemstrømningen og glukosemetabolismen i forlårsmuskulaturen hos patienter med type 2 diabetes og matchede kontrolpersoner – effekten af 8 ugers kontrolleret høj intens intervaltræning*

I forbindelse med, at vi skal i gang med at afvikle et videnskabeligt forsøg, søger vi forsøgspersoner. Vi vil i denne forbindelse spørge dig om du vil deltage i forsøget, der udføres af lektor Per Bendix Jeppesen, cand. scient., ph.d.-studerende Anne Cathrine Thorup og ph.d.-studerende Søren M. Madsen.

Før du beslutter dig for om du ønsker at deltage i forsøget, vil vi gerne gøre dig opmærksom på forsøgets indhold og hvorfor vi gerne vil gennemføre dette forsøg. Derfor ønsker vi, at du er omhyggelig med at læse og forstå indholdet i denne deltagerinformation.

Du inviteres til en samtale om forsøget. Her vil deltagerinformationen blive uddybet og såfremt du i denne forbindelse måtte være i tvivl om noget, kan du frit stille de spørgsmål, du måtte have om forsøgets indhold. Du er velkommen til at medbringe et familiemedlem, kæreste, ven eller bekendt til samtalen.

Såfremt du er indstillet på at deltage i forsøget, vil vi bede dig om at underskrive en samtykkeerklæring. Før du beslutter dig for om du vil underskrive samtykkeerklæringen, har du naturligvis ret til betænkningstid. Du skal være opmærksom på, at det er frivilligt at deltage i forsøget. Heri ligger også, at du når som helst og uden at give en grund kan trække dit samtykke tilbage (se evt. detaljer i samtykkeerklæringen).

## Formål med forsøget

Det overordnede formål med nærværende studie er at undersøge blodgennemstrømningen i forlårsmuskulaturen og sukkerstofskiftet før og efter 8 uger med høj intens intervaltræning (HIIT) på cykelergometer hos patienter med type 2 diabetes (T2D) og matchede kontrolpersoner. Følgende parametre indgår i vurderingen heraf:

- Sukkerstofskiftet
- Muskel- og fedtmassen
- Blodgennemstrømningen i hvile omkring forlårsmuskulaturen
- Iltoptagelsestest på cykelergometer samt 5 km tidskørsel

**Baggrund for forsøget**

T2D er blandt verdens hurtigst voksende livsstilsygdomme. Alene i Danmark er der ifølge Sundhedsstyrelsen ca. 245.000 diagnosticerede T2D-patienter. Hertil kommer, at det er estimeret, at lige så mange menes at have T2D uden at have kendskab til det. Derudover har op til 750.000 danskere begyndende diabetiske komplikationer. T2D udgør også en sundhedsmæssig risiko i resten af verden; i 2010 mentes 285 millioner at have T2D, og dette forventes at øge eksplosivt med op til 439 millioner i 2030. Dermed fremstår T2D som en af det 21. århundredes største globale sundhedsudfordringer.

T2D skyldes en kombination af en tilstand, hvor der er behov for mere insulin end normalt for at udløse et normalt biologisk respons (perifer insulinresistens) og forstyrrelser i de insulinproducerende celler i bugspytkirtlen, hvilket især giver anledning til lavere glukoseoptag i skeletmuskulaturen sammenlignet med ikke-type 2 diabetiske individer. Genetik, fedme og fysisk inaktivitet er tre vigtige faktorer, der ligger til grund for disse metaboliske forstyrrelser. Samtidig med udviklingen af T2D, kan de nævnte metaboliske forstyrrelser (perifer insulinresistens og forstyrrelser i de insulinproducerende celler i bugspytkirtlen) også give anledning til at udvikle en række andre hjerte-kar-sygdomme, herunder f.eks. forøget blodtryk, blodprop i hjertet, småkarssygdomme (kapillærer) i organer og ben. Foruden ovenstående T2D karakteristika, menes sygdommen også at give anledning til ændret – og i nogle tilfælde nedsat – blodgennemstrømning i musklerne, hvilket kan skyldes en ændring af beskaffenheden af blodkarrenes indre cellelag, såkaldt endotelceller. Hvis endotelcellerne ikke er i stand til at ’svare’ passende på et signal (f.eks. træning eller mekanisk ændring ved brug af blodtryksmanchet) er det udtryk for, at karrene er mindre gode til at udvide sig, hvilket betinger en dårligere iltoverførsel til muskelvævet og skyldes formentlig åreforkalkning af endotelcellerne (endothelial dysfunktion) som følge af T2D-sygdommen. Denne tilstand kan måles i hvile ved en metode kaldet flowmedieret vasodilatation (FMD). Det er en non-invasiv procedure (dvs. kroppen udsættes ikke for indgreb), hvor der anvendes ultralyd og en blodtryksmanchet med henblik på at vurdere karrenes evne til at udvide sig efter, at blodtryksmanchetten har været pustet op i 5 minutter.

I et for nylig publiceret studie er det blevet vist, at patienter med øget blodtryk har lavere blodgennemstrømning under et-bens sparkearbejde sammenlignet med forsøgspersoner med normal blodtryk. Hvorvidt blodgennemstrømningen (ved brug af FMD) ændres hos T2D patienter efter længere tids høj intens træning vides ikke, men er bl.a. blevet undersøgt hos individer med hjertesygdom, hvor karrenes evne til at udvise blev øjeblikkelig forbedret efter høj intens træning én gang.

Der er ikke enighed om, hvilken specifik træningsstrategi, der er nødvendig for at tilvejebringe ovenstående helbredsfordele for T2D patienter; i særdeleshed med hensyn til forholdet mellem volumen og intensitet. Høj intens intervaltræning (HIIT) er en træningsstrategi, som omfatter korte anstrengende omgange, hver individuelt efterfulgt af en restitutionsperiode, der er tilstrækkelig lang til, at forsøgspersonen er i stand til at komme sig med henblik på at kunne udføre tilsvarende maksimale anstrengelse efterfølgende. I den forbindelse er det blevet forsøgt at udvikle en HIIT protokol til fordel for T2D patienter og stillesiddende individer. HIIT-protokollen omfatter 10×60 sekunders intervaller afbrudt af 60 sekunders pause mellem hvert interval, og med en arbejdsintensitet svarende til en kraft, der under en iltoptagelsestest fremkalder ca. 90% af den maksimale hjertefrekvens. Så vidt vides er der to forskningsgrupper, som har testet denne træningsform på henholdsvis T2D patienter og stillesiddende individer, begge udført over to uger med betydelige metaboliske effekter, herunder især mindre blodsukker. Dette kan indikere, at HIIT-strategi kan medføre væsentlige metaboliske effekter, herunder reducere den diabetiske tilstand og dermed give anledning til bedre helbred for denne patientgruppe.

**Forsøgsoversigt og metoder**

Forsøgets formål er at vurdere de mulige helbredsmæssige fordele af 8 ugers HIIT-forløb. Umiddelbart før og efter HIIT-forløbet møder du ind til i alt 7 undersøgelser, der skal vurdere dit helbred ud fra forskellige målinger. De omtalte målinger vil nu blive uddybet:

- Maksimal iltoptagelsestest på cykelergometer på Sektion for Idræt, Aarhus Universitet. Højde, vægt og blodtryk registreres. Hertil kommer, at der udføres elektrokardiogram af dit hjerte før og efter cykel-testen. Et pulsbælte sættes omkring dit bryst, hvorpå opvarmningen (8-10 min) indledes. Herefter sættes maske omkring dit ansigt, hvorpå cykel-testen begynder. Startintensitet er 50 watt med en selvvalgt omdrejningsfrekvens, og belastningen stiger med 15 watt pr. minut frem til udmattelse.
- Tidskørsler foregår på Sektion for Idræt, Aarhus Universitet. Tidskørsel (5 km) udføres på cykelergometer, hvoraf den hurtigste bruges til senere sammenligning. Ingen feedback gives, på nær antal kørte km. Anden tidskørsel udføres to dage efter den første; den hurtigste tilbagelagte tid af de to tidskørsler bruges til senere sammenligning.
- Sukkerbelastningstest (oral glukose tolerance test (OGTT)): du møder ind fastende på Endokrinologisk Afdeling (Medicinsk Afd. M), Aarhus Universitetshospital. Du vil få anlagt et venflon af en læge eller sygeplejeske i albueleddet, hvorefter der vil blive taget blodprøver (ca. 5 ml pr. blodprøve) 30 og 15 minutter før OGTT samt til tiden 0 (umiddelbart efter drikkes en sukkeropløsning), 10, 20, 30, 45, 60 og 120 min, hvorefter testen afsluttes.
- Måling af kropssammensætning (DXA-skanning): foretages i forlængelse af OGTT, og foregår på Endokrinologisk Afdeling (Medicinsk Afd. M), Aarhus Universitetshospital. Med en DXA-skanner kan man måle din fedtfri masse, fedtmasse og kropsvægt. Skanningen er uden ubehag og varer ca. 15 min, og foretages henholdsvis før og efter træningsinterventionen.
- Blodgennemstrømningsmåling i hvile ved brug af ultralydsskanner og blodtryksmanchet før og efter maksimal iltoptagelsestest og foregår på Sektion for Idræt, Aarhus Universitet. Samme forløb gentages efter HIIT-forløbet. Testen foregår således, at du placeres på en briks og hviler dig ≥ 20 minutter. Der måles indledningsvist 1 minut i hvile, hvorefter manchetten pustes op omkring forlårsmuskulaturen i 5 minutter (hvor der skannes) efterfulgt af en sluttelig skanning på 5 minutter. Du skal være fastende forud for forsøget. Det er også vigtigt, at du holder pause med kaffe, the, cola og chokolade mindst 24 timer før undersøgelsen, da det kan påvirke målingen af blodgennemstrømningen. Ligeledes er det vigtigt, at du ikke har trænet mindre end 24 timer forud for forsøget.

*Træningsprogram*

Du udfører superviseret højintens intervaltræning 3 gange om ugen over 8 uger på cykelergometer (med wattmåler) og lokale stilles til rådighed hos et lokalt fitnesscenter. Forud for den 8 uger lange træningsperiode møder du ind til en gennemgang af cyklens opsætning, hvor du får mulighed for at vænne dig til cyklen (herunder belastning, højde- og styrindstilling). Under træningsperioden udfører du et individuelt fastlagt program, som hver gang omfatter 10×60 sek. arbejde (afbrudt af 60 sek. pause) svarende til en kraft, der under cykel-testen fremkalder ~90% af den maksimale hjertefrekvens. Tidsforbruget per session forventes at vare ~25 minutter.

## Plan for forsøget

Udfyldning af samtykkeerklæring.

Da der i forbindelse med forsøget anvendes flere metoder (anlæggelse af katetre, DXA- og ultralydsskanninger (med og uden kontraststof) samt sukkerbelastningstests) vil du inden forsøget blive bedt om at udfylde en samtykkeerklæring, som forsøgslederen efterfølgende gennemgår med dig. Det sker for at sikre, at du er indforstået med, hvad der skal ske. Der er beskrevet meget få bivirkninger ved de ovenfor nævnte metoder.

## Nytte ved forsøget

Din eventuelle deltagelse i dette forsøg har en vigtig forskningsmæssig nytte, idet din deltagelse er med til at forstå reguleringen af blodgennemstrømningen i store og mindre kar, både i hvile og under fysisk aktivitet. Hvis vore antagelser om forstyrret blodgennemstrømning hos T2D patienter viser sig at være rigtige, vil vi på sigt kunne bruge vore erfaringer til at udvikle et diagnostisk værktøj, der kan benyttes til udredning af mange hjerte/kar relaterede sygdomme. Der er dog ingen behandlingsmæssig gevinst ved at deltage i forsøget.

## Bivirkninger, risici, komplikationer og ulemper

Forsøget er ikke forbundet med nogen kendte risici eller gener.

*DXA-skanninger*

I forbindelse med DXA-skanningen er strålebelastningen relativ beskeden svarende til 0.02-0.05 millisievert pr. undersøgelse (dvs. i alt 0.04-0.10 millisievert i forsøget). Til sammenligning er den årlige baggrundsstråling i Danmark 3.0 millisievert. I Danmark er risikoen for at få kræft 25%. Med skanningerne i dette forsøg svarer det til, at der er 25.0005% øget risiko for kræft (dvs. 0.0005%).

*Sukkerbelastningstest*

Der er minimale gener forbundet med blodprøveudtagning, og da der gennem hele forsøgsperioden udtages omtrent 100 ml blod (ca. 1/4 af en almindelig bloddonation), udgør dette derfor ingen risiko for forsøgspersonen. Venflonanlæggelse foregår under sterile forhold. Du rådes til at undgå intenst fysisk aktivitet om aftenen på selve forsøgsdagen. En mindre blodansamling som følge af venflonanlæggelsen kan forekomme og give ømhed og gener i dagene umiddelbart efter forsøget.

Samlet set vurderes det derfor, at eventuelle risici og bivirkninger opvejes af de forventelige fordele ved deltagelse i forsøget.

**Inklusions- og eksklusionskriterier**

For inklusionskriterierne gælder det, at forsøgsdeltagerne i forsøget er T2D patienter eller matchede kontrolpersoner hertil. I alt ansøges der om at inkludere 34 forsøgspersoner (17 T2D patienter og 17 kontrolpersoner). Inklusionskriteriet for T2D i dette studie er en glukosekoncentration af venøs plasma ≥ 11.1 mmol/L 2 timer efter OGTT. Desuden gælder det, at samtlige forsøgspersoner har en alder mellem 40-60 år og et body mass index mellem 25-40 kg/m^2^. Køn er underordnet.

Mulige forsøgsdeltagere kan ekskluderes, såfremt de har ustabil hjertesygdom eller alvorlig lungesygdom. Ydermere ekskluderes du, hvis du er gravid eller ammer eller er kraftig overvægtig (BMI>40 kg/m^2^). Desuden må du ikke være undersøgt med eller have deltaget i andre forsøg, der involver ioniserende stråling indenfor det sidste år. Hertil kommer, at insulinbrug og moderat intensitetstræning i mere end 1 time pr. uge ekskluderer din deltagelse.

Forsøget afbrydes ved mindste tegn på utilpashed samt hvis du ikke kan udføre de fysiske tests.

## Økonomisk vederlag til forsøgsdeltagere

Der ydes dog et honorar på i alt 3000 kr. til forsøgspersonerne, som opgives til SKAT som B-indkomst. Ydermere godtgøres (2.10 kr/km) der for transporten til og fra forsøgsstederne, såfremt patienten kommer langt væk fra Århus. Desuden dækker Aarhus Universitetshospital for omkostningerne til deltagernes træning i et lokalt træningscenter.

Forsøgsdeltagerne hverves på baggrund af et rekrutteringsopslag, der vil blive hængt op på sundhedscentre i Region Nordjylland og Regions Sønderjylland, på Aarhus Universitetshospital og i aviserne Århus Onsdag og Nordjyske. Samt på [www.forsøgsperson.dk](http://www.forsøgsperson.dk).

## Økonomisk støtte

Der vil blive søgt støtte fra Diabetesforeningen, Hjerteforeningen og Århus Universitets frie forskningsmidler. Hertil kommer, at der også forsøges at blive søgt hos andre relevante fonde.

**Forsikring**

Patienterne dækkes i henhold til patientforsikringsloven.

**Offentliggørelse**

Dette forsøg danner grundlaget for Søren Møller Madsens ph.d.-projekt. I den forbindelse vil publikation af resultater fra projektet koordineres af den projektansvarlige i overensstemmelse med retningslinjer angivet i ’Vejledninger for god Videnskabelig Praksis’, Udvalget Vedrørende Videnskabelig Uredelighed, 1998. Positive såvel som negative resultater af undersøgelserne vil løbende blive offentliggjort i internationale videnskabelige tidsskrifter, f.eks. Journal of Physiology.

Det er vores ønske med denne deltagerinformation, at du har fået tilstrækkelig information om deltagelsen i forsøget, og at du føler dig rustet til at tage beslutningen om din potentielle deltagelse. Vi vil også bede dig om at læse det vedlagte materiale: ’Forsøgspersonens rettigheder i et biomedicinsk forskningsprojekt’.

Under ledelse af lektor Per Bendix Jeppesen (MEA):

Per Bendix Jeppesen

Medicinsk Endokrinologisk Afdeling, Aarhus Universitet

Aarhus Universitetshospital

Tage Hansens Gade 2

8000 Aarhus C.

Telefon: 28 15 18 77

E-mail: [per.bendix.jeppesen@ki.au.dk](mailto:per.bendix.jeppesen@ki.au.dk)

Ønsker du at vide mere om forsøget, kan du altid kontakte:

Søren Møller Madsen

Medicinsk Endokrinologisk Afdeling, Aarhus Universitet

Aarhus Universitetshospital

Tage Hansens Gade 2

8000 Aarhus C.

Telefon: 61 67 17 91

E-mail: [smmadsen@cfin.dk](mailto:smmadsen@cfin.dk)


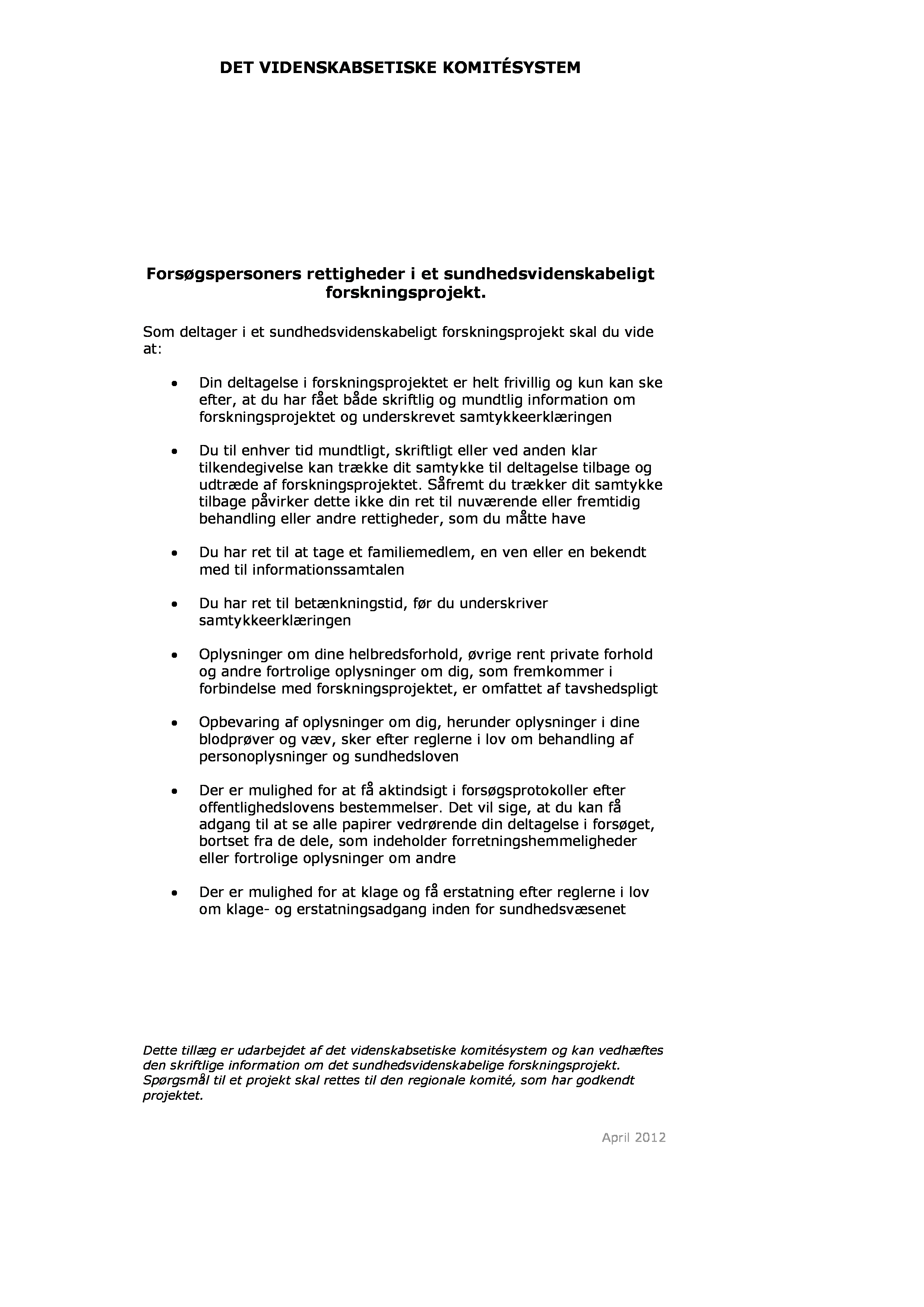


Da der ikke har været mulighed for at kopiere samtykkeerklæringen, informeres der hermed om, at S1-dokumentet vil blive anvendt.
